# Phosphorus starvation response and PhoB-independent utilization of organic phosphate sources by *Salmonella enterica*

**DOI:** 10.1128/spectrum.02260-23

**Published:** 2023-10-03

**Authors:** Roberto E. Bruna, Christopher G. Kendra, Mauricio H. Pontes

**Affiliations:** 1 Department of Pathology and Laboratory Medicine, Pennsylvania State University College of Medicine, Hershey, Pennsylvania, USA; 2 The One Health Microbiome Center, Huck Institute of the Life Sciences, Pennsylvania State University, University Park, Pennsylvania, USA; State Key Laboratory of Microbial Resources, Institute of Microbiology, Chinese Academy of Sciences, Beijing, China

**Keywords:** PhoB/PhoR, organic phosphate, phosphorus, stress response, RNA-seq, *Salmonella*

## Abstract

Inorganic orthophosphate (Pi, PO_4_^3−^) is the preferred phosphorus (P) source of most living cells. In enteric bacterial species such as *Salmonella enterica* serovar Typhimurium (*Salmonella*), removal of Pi from the growth medium activates a dedicated two-component signal transduction system which is composed of the membrane-bound sensor kinase PhoR and its cognate transcriptional regulator PhoB. Activated PhoB promotes transcription of genes that are involved in adaptations to low Pi conditions, such as high-affinity Pi transporters and genes required for the assimilation of alternative P sources, allowing bacteria to adapt to low Pi environments. Here, we characterize the PhoB-dependent and independent transcriptional changes elicited by *Salmonella* in response to P starvation. We identify a set of organic molecules that can serve as the sole P source and pinpoint genes that are involved in their assimilation. Intriguingly, a number of these genes are not under PhoB/PhoR control, indicating that in the absence of environmental Pi, *Salmonella* may fulfill its biosynthetic demands for P without activating a transcriptional response to Pi starvation.

**IMPORTANCE:**

Phosphorus (P) is the fifth most abundant element in living cells. This element is acquired mainly as inorganic phosphate (Pi, PO_4_^3−^). In enteric bacteria, P starvation activates a two-component signal transduction system which is composed of the membrane sensor protein PhoR and its cognate transcription regulator PhoB. PhoB, in turn, promotes the transcription of genes that help maintain Pi homeostasis. Here, we characterize the P starvation response of the bacterium *Salmonella enterica*. We determine the PhoB-dependent and independent transcriptional changes promoted by P starvation and identify proteins enabling the utilization of a range of organic substrates as sole P sources. We show that transcription and activity of a subset of these proteins are independent of PhoB and Pi availability. These results establish that *Salmonella enterica* can maintain Pi homeostasis and repress PhoB/PhoR activation even when cells are grown in medium lacking Pi.

## INTRODUCTION

Phosphorous (P) is an integral constituent of various biological molecules. This element is a structural component of phospholipids, lipopolysaccharides, and (lipo)theichoic acid polymers and, consequently, is required for the formation of membranes and other structures that make up cellular envelopes and intracellular organelles. As a component of nucleotides and sugar phosphates, P participates in cellular metabolism, energy, and information transduction, and serves as a building block for various macromolecules, including (deoxy)ribonucleic acids (1, 2) which store and mediate the expression of genetic information. How do bacterial cells maintain P homeostasis?

In *Escherichia coli, Salmonella enterica* serovar Typhimurium (*Salmonella*), and closely related bacterial species, P is assimilated primarily through the consumption of inorganic orthophosphate (Pi, PO_4_^3−^). Typically, the acquisition of environmental Pi relies on the activity of proteins belonging to the Pit (phosphate inorganic transport) family, such as PitA, and a transporter composed of the PstSCAB proteins (1, 2). PitA-type transporters use the proton motive force to translocate neutral metal:Pi salts across the cytoplasmic membrane. Because of their low substrate affinity, these proteins usually serve as housekeeping transporters (1 – 5). In contrast, PstSCAB is an inducible, high-affinity, ATP-dependent transporter that is expressed in response to Pi starvation. PstSCAB is encoded in the *pstSCAB-phoU* operon, which is transcriptionally activated by the PhoB/PhoR two-component signal transduction system (1, 2, 6).

PhoR is a membrane-bound bifunctional histidine kinase/phosphatase and PhoB is its cognate response regulator. Pi limitation promotes PhoR kinase activity. PhoR auto-phosphorylates and subsequently transfers the phosphoryl group to an aspartic residue on PhoB (7, 8). PhoB-P binds to DNA sequences (Pho boxes) in the promoter region of the Pho-regulon genes and modulates their transcription (9 – 12). Members of the Pho regulon with known functions have been historically characterized in *E. coli* and include those directly involved in Pi signaling and transport (PhoB/R, Pst syst, PhoE) and the Pi extraction from alternative organic-P sources (e.g., *phn* and *ugp* genes) (1, 6, 7). Although *Salmonella* is expected to share core members of the Pho regulon with *E. coli* (13), its physiological response to P limitation has not been systematically characterized.

In this study, we used RNA-seq to define the transcriptional response of *Salmonella* to P starvation. We compare the transcriptional profiles of wild-type and *phoB* mutant strains and, through an *in silico* analysis, we identify putative PhoB-binding sites upstream of 28 previously uncharacterized PhoB-dependent transcriptional units. We establish that *Salmonella* can use a variety of organic-P compounds as a sole P source, including substituted phosphonates and phosphate esters. We identify the genes required for the utilization of these substrates, and we determine whether their expression and activity are PhoB-dependent. Our findings provide a global view of the *Salmonella* transcriptional response to P limitation and identify a group of PhoB-independent genes that mediate the utilization of organic-P sources.

## RESULTS

### Overview of the transcriptomic response of *Salmonella* to P starvation

When exponentially growing *Salmonella* undergoes a nutritional downshift from Pi-rich medium to medium lacking a P source, PhoB/PhoR undergoes a rapid activation surge: fluorescence derived from plasmid-borne transcriptional fusions between the PhoB-activated genes *phoB* or *pstS* and an unstable green fluorescence protein (*gfp*_AAV_) peaked at approximately 35 min following the downshift (Fig. S1A) (14). In contrast, the activities of these fluorescent fusions did not change considerably when, instead, cells were shifted to a fresh Pi-rich medium (Fig. S1A). Both the wild-type and *phoB* mutant strains of *Salmonella* grew at similar rates, reached equivalent growth yields, and retained equal viability after 35 min in medium lacking P (Fig. S1B and C). Therefore, failure to activate PhoB neither impaired growth nor affected bacterial viability during P starvation (15 – 18).

**Fig 1:**
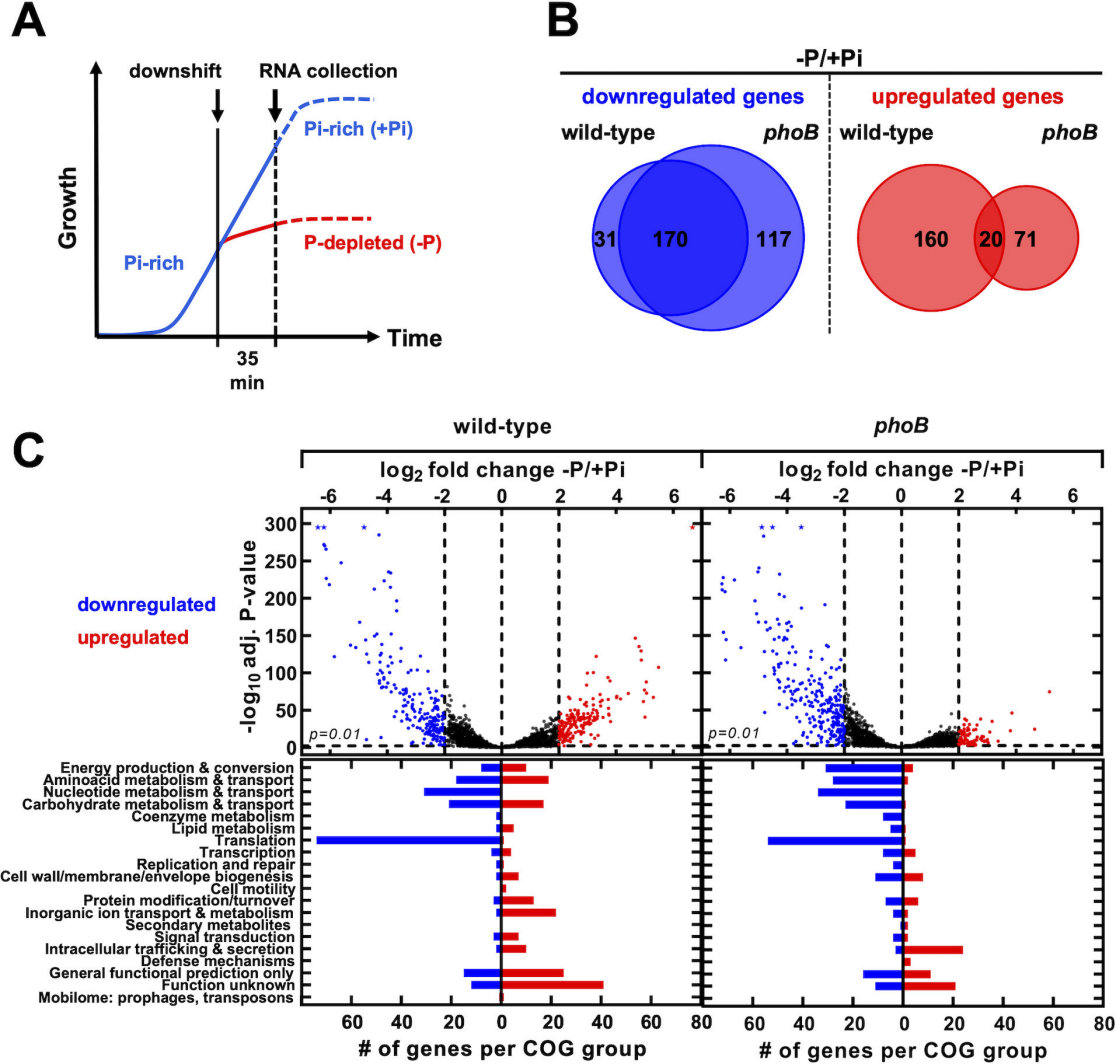
Overview of *Salmonella* transcriptomic response to P starvation. (A) RNA-seq experimental design. Wild-type (14028s) and *phoB* mutant (EG9054) *Salmonella* cells were grown to mid-exponential phase in Pi-rich (+Pi, 1 mM K_2_HPO_4_) MOPS medium. At this point, cultures were split in halves and either subjected to a P-starvation treatment (-P) or maintained in a Pi-rich (1mM K_2_HPO_4_) MOPS medium (+Pi). Growth continued for anadditional35min, before proceeding to RNA extraction. Further experimental details are described in theMaterials and Methods. (B)Quantitative Venn diagrams representing genes whose corresponding mRNAs are at leastfourfolddifferent (log2fold change>2) between−P and+Pi treatments. (C)(Top) Volcano plots depicting the expression fold changeversusthe−log10of the corresponding adjusted*P*-value for all genes between−P and+Pi conditions in wild-type and*phoB*mutant strains. Blue dots represent genes exhibitinga≥4-folddecrease in transcripts, whereas red dots represent genes exhibitinga≥4-foldincrease in transcripts between conditions. Asterisks (*) represent transcripts with clipped values: logs of very small adjusted*P*-values are represented as 295. Data correspond to DESeq2 expression analysis offourbiological replicates using Geneious Prime software. (Bottom) Frequency distribution of assigned COG categories for genes exhibitinga≥4-folddecrease (blue bars) or increase (red bars) in expression between−P and+Pi treatments.

We used RNA-seq to identify PhoB-dependent and independent transcriptional changes elicited by P starvation in *Salmonella*. We compared the transcriptomic profiles of exponentially growing wild-type and *phoB* mutant strains at 35 min following shifts from Pi-rich medium to either medium lacking a P source or fresh Pi-rich medium (Fig. 1A). In wild-type cells, P starvation caused the upregulation of 180 genes and the downregulation of 201 genes (≥4-fold change, adjusted *P*-value < 0.01). In comparison, 91 and 287 genes were upregulated and downregulated, respectively, in *phoB* mutant cells (Fig. 1B and C; Table S1). In both wild-type and *phoB* mutant strains, upregulated transcripts were overrepresented within cluster of orthologous genes (COG) categories encoding proteins with unknown and general function (Fig. 1C). Prominently, P starvation caused a PhoB-independent increase in the transcription of genes within *Salmonella* pathogenicity island-2 (SPI-2) (Fig. 1C; Fig. S2 and Table S1) (18 – 22). This horizontally acquired chromosomal region encodes several virulence proteins, including the structural components of a type III secretion system that is required for *Salmonella* replication within mammalian macrophages (23 – 27).

**Fig 2:**
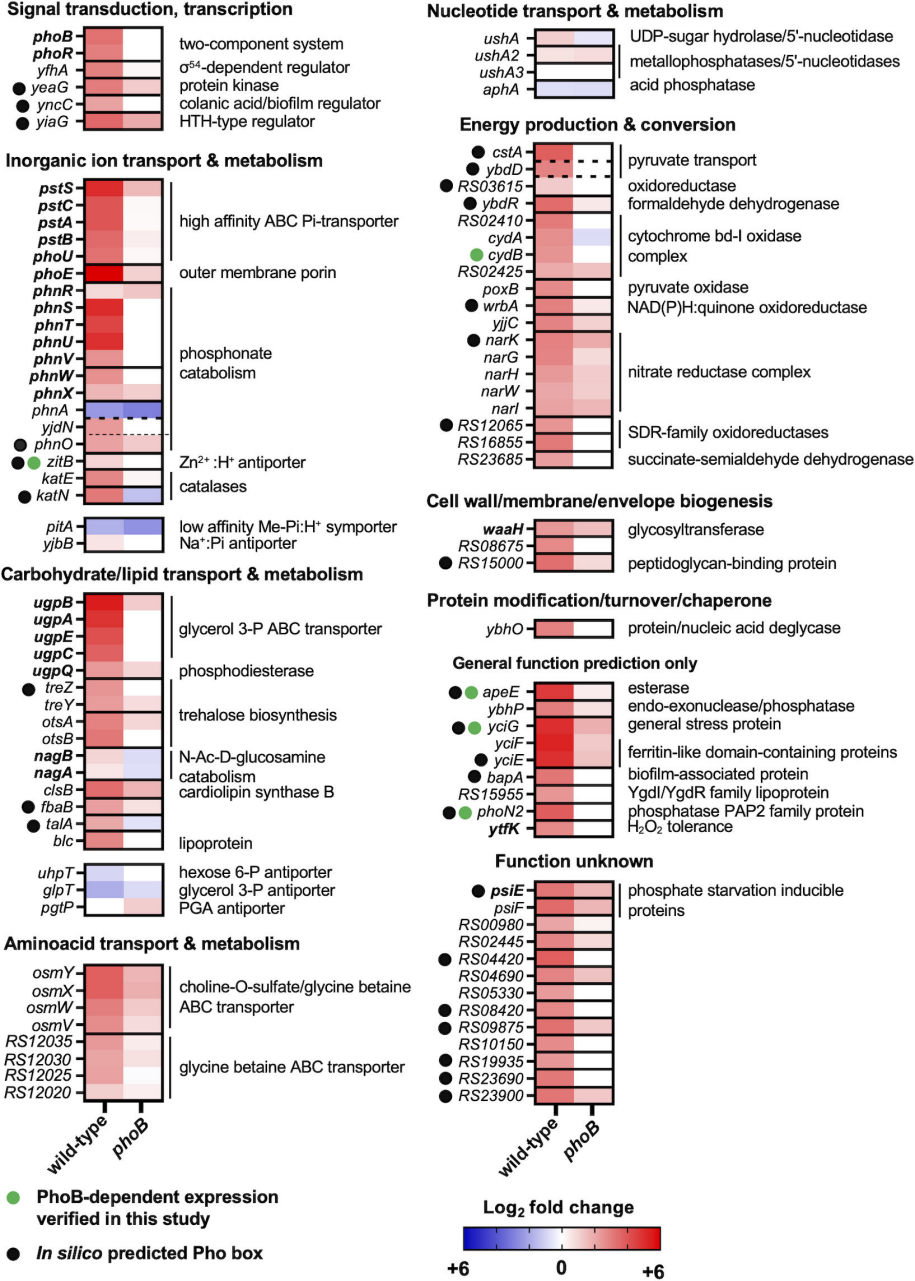
PhoB-dependent and transcriptional changes during *Salmonella* P starvation. Heatmaps depicting fold changes in transcript levels between −P and +Pi treatments for wild-type (14028s) and *phoB* mutant (EG9054) strains of *Salmonella*. Graphs show transcripts from genes with the highest fold change between wild type and *phoB* during P starvation (log_2_ < −3; Tables S1 and S2). Genes are grouped by COG categories. Genes in different transcriptional units are boxed by solid lines, while genes located contiguously in the genome but belonging to separate transcriptional units are marked with non-contiguous lines. A subgroup of PhoB-independent P-acquisition-related genes is depicted. Members of the Pho regulon that have been identified in previous studies (either in *E. coli* or *Salmonella*) for which direct PhoB regulation has been confirmed by electrophoretic mobility and/or DNA footprinting assays are labeled in bold. Genes whose PhoB-dependent upregulation during P starvation was experimentally verified in this study (Fig. 2) are labeled with ●. Genes in which putative PhoB-binding motifs were identified *in silico* in this study are labeled with ●. Note that NCBI gene locus tags from the 14028s genome are abbreviated (e.g., locus tag *STM14_RS12020* is simply displayed as *RS12020*).

**Fig 3:**
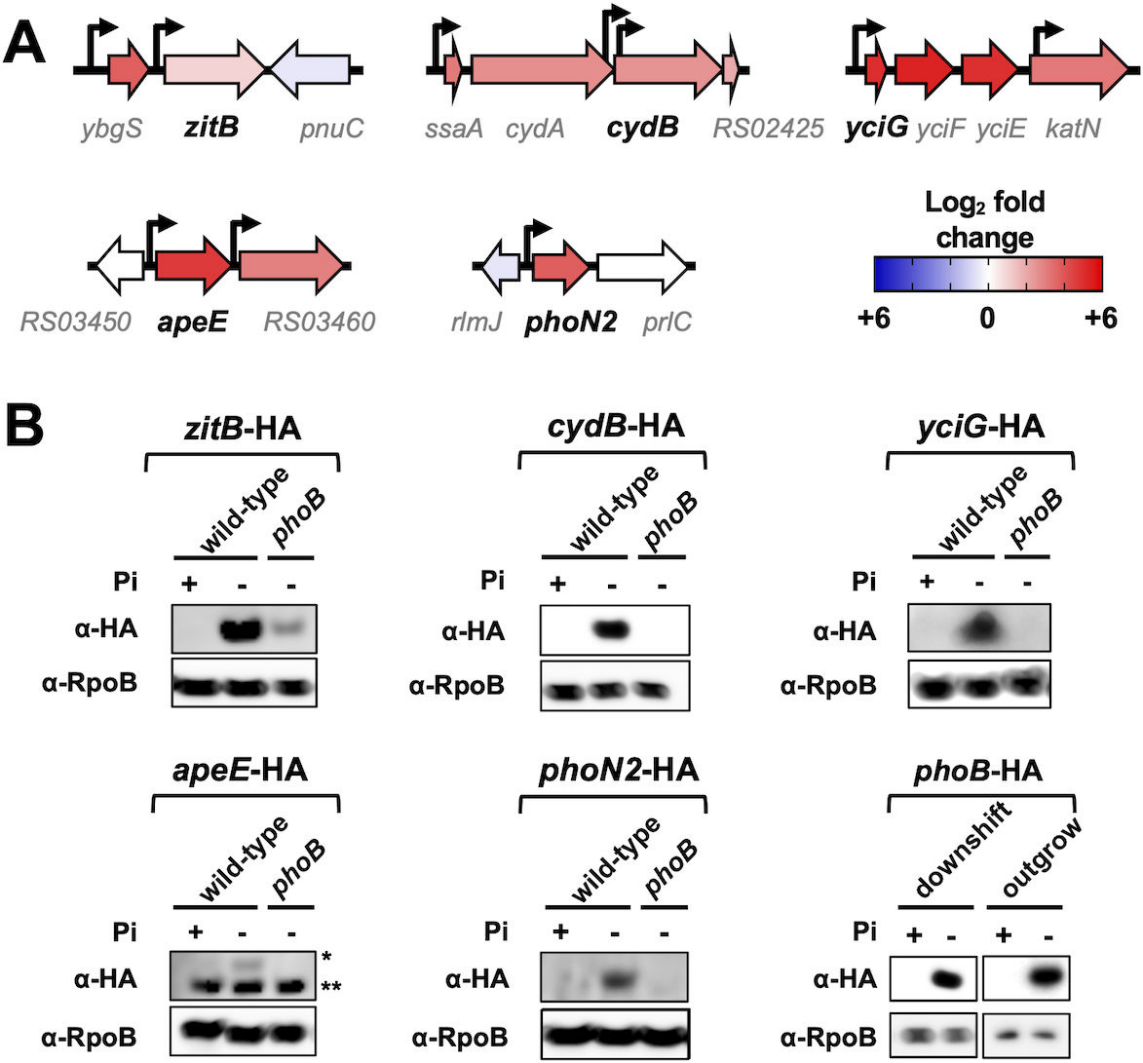
Verification of RNA-seq results. (A) Diagrams depicting the genomic context of *zitB* (*STM14_RS04415*), *cydB* (*STM14_RS02420*), *yciG* (*STM14_RS09540*), *apeE* (*STM14_RS03455*), and *phoN2* (*STM14_RS19030*) loci in *Salmonella*. The bent arrows represent previously mapped transcriptional start sites (20, 28). Protein-coding sequences are represented by large arrows, colored according to their fold-change expression in wild-type cells subjected to P starvation (Fig. 2; Tables S1 and S2). Note that NCBI gene locus tags from the 14028s genome are abbreviated (e.g., locus tag *STM14_RS02425* is simply displayed as *RS02425*). (B) Western blot analysis of protein extracts prepared from wild-type (14028s), *phoN2*-HA (RB441), *phoB phoN2*-HA (RB447), *yciG*-HA (RB439), *phoB yciG*-HA (RB445), *apeE*-HA (RB440), *phoB apeE*-HA (RB446), *zitB*-HA (RB437), *phoB zitB*-HA (RB443), *cydB*-HA (RB438), *phoB cydB*-HA (RB444), and *phoB*-HA (MP1429) strains. Protein extracts were obtained from cultures that were either starved for P or grown in Pi-rich medium. For PhoN2-HA and YciG-HA detection, exponentially grown cells were subjected to a 35-min long downshift to MOPS medium lacking a P source. For ApeE-HA, ZitB-HA, and CydB-HA, P starvation was achieved by outgrowing cultures in MOPS medium containing 50 µM K_2_HPO_4_ during 18 h. ApeE-HA is indicated by a single asterisk (*) and the the lower band (indicated by **) is a non-specific loading control. Control cells were cultured in MOPS medium containing 1 mM K_2_HPO_4_ for the duration of the experiment. Because PhoB promotes its own transcription (29), detection of PhoB-HA was used as a P starvation control. Detection of RpoB was used as a loading control. Images are representative of three independent experiments.

P starvation downregulated 170 transcripts in both wild-type and *phoB* mutant cells (Fig. 1B and C; Table S1). Downregulated RNA species were overrepresented in COG categories of proteins involved in ribosomal structure, biogenesis and translation (i.e., ribosomal proteins, ribosomal maturation factors, elongation, and release factors), nucleotide transport and metabolism (i.e., purine, pyrimidine, nucleoside, and nucleotide biosynthetic pathways), and amino acid transport and metabolism (i.e., polyamine biosynthesis) (Fig. 1B and C; Fig. S2 and Table S1). Curiously, many of these genes are transcriptionally repressed by the alarmone (p)ppGpp (28, 30 – 33). During P starvation, (p)ppGpp accumulates as a result of the inhibition of the hydrolytic activity of the bifunctional (p)ppGpp synthase/hydrolase SpoT (34 – 36). Because (p)ppGpp promotes the transcription of SPI-2 genes (28, 37 – 39), the effect of P starvation on SPI-2 transcription (Fig. S2) may result from changes in SpoT’s activity.

P starvation also increased transcripts from 160 genes in the wild type but not in the *phoB* mutant strain (Fig. 1B and C; Table S2; ≥4-fold change, adjusted *P*-value < 0.01). Most of these genes belong to COG categories encoding proteins involved in energy production and conversion, carbohydrate, amino acid, and inorganic ion transport and metabolism (Fig. 1C and 2). These include several members of the PhoB regulon that were previously characterized in *E. coli* (i.e., *phoBR*, *phoE*, *pstSCAB-phoU*, *ugpBAECQ*, *phnXWRSTUV*, and *psiE*) (1, 6) and *Salmonella* (i.e., *apeE*, *nagA*, and *nagB*) (18, 40) (Fig. 2).

Because a substantial fraction of transcription start sites has been inferred in *Salmonella* (20, 28), we used the web-based MEME Suite (41) to search for Pho boxes within or at the vicinity of the promoter regions of the aforementioned PhoB-dependent transcripts (10 42). Putative Pho boxes were identified upstream of 28 transcriptional units (Fig. 2; Fig. S3 and Tables S3 and S4), suggesting that downstream gene(s) may be under direct PhoB control. Taken together, these results indicate that P starvation triggers a rapid transcriptional reprogramming in *Salmonella*. Whereas most of the observed changes reflect PhoB-independent repression of transcripts that are often associated with cell growth (e.g., stringently repressed genes), most upregulated transcripts are PhoB-dependent.

### PhoB-dependent expression of ZitB, CydB, YciG, ApeE, and PhoN2 during P starvation

We sought to measure the effect of P starvation on protein levels of five PhoB-activated genes: *zitB*, *cydB*, *yciG, apeE*, and *phoN2* (Fig. 2, 3A; Table S2). To this end, we engineered a set of *Salmonella* strains, each harboring an in-frame hemagglutinin (HA) epitope tag at their 3′ end. Logarithmically growing, HA-tagged wild-type and *phoB* mutant cells were subjected to a downshift from Pi-rich medium to medium lacking P, and protein levels were assayed by Western blot. We determined that P starvation increased the amounts of ZitB-, CydB-, YciG-, ApeE-, and PhoN2-HA in the wild type but not in the *phoB* mutant strain (Fig. 3B). These results independently verify part of the transcriptional data presented above (Fig. 2; Table S2).

### PhoB-dependent genes support the growth of *Salmonella* on 2-aminoethylphosphonate and *sn*-glycerol-3-phosphate as sole P source

**Fig 4:**
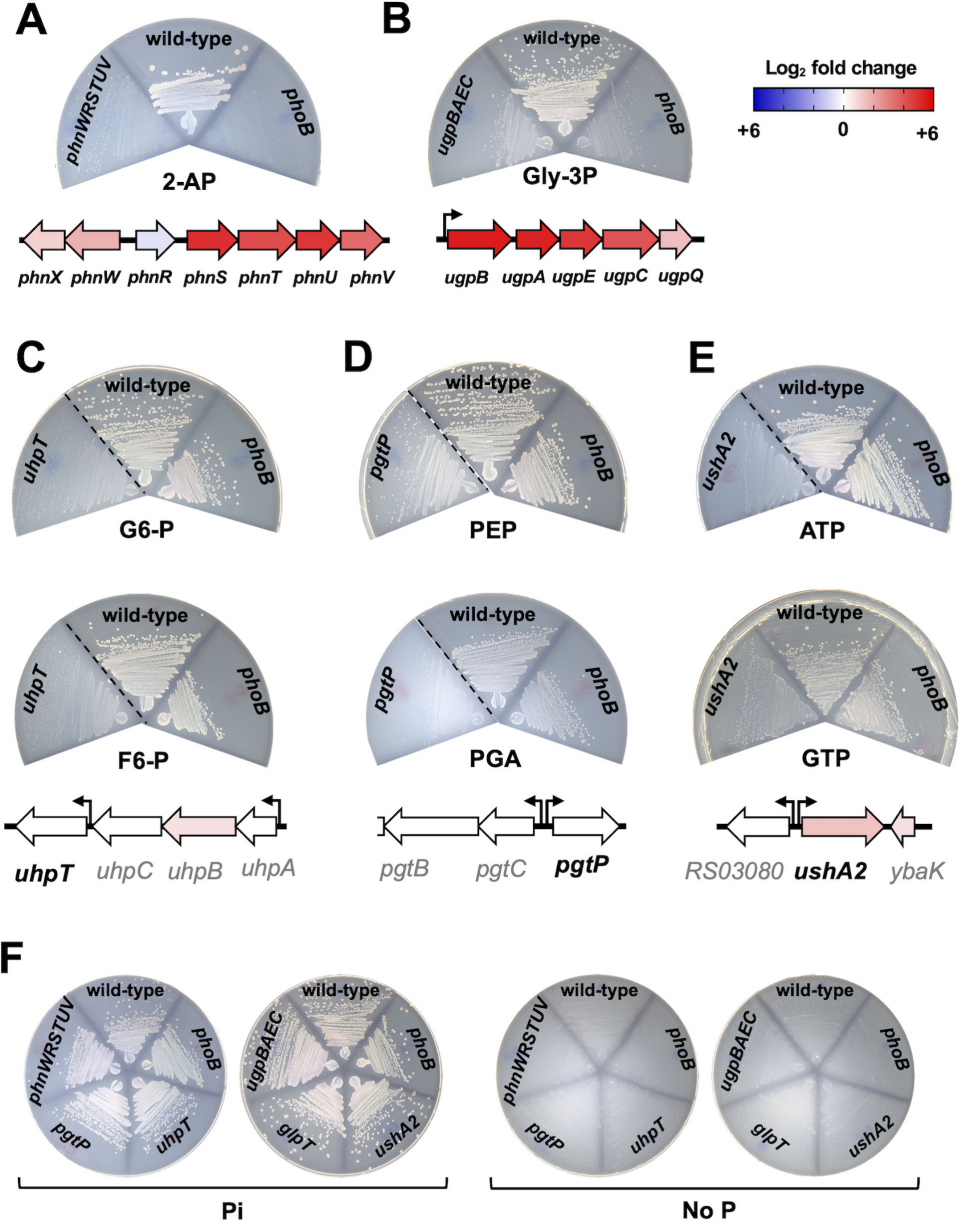
Genetic requirements of *Salmonella* for growth on different organic compounds as the sole P source. Wild-type (14028s), *phoB* (EG9054), *ugpBAEC* (MP1736), *glpT* (MP1737), *phnWRSTUV* (MP1784), *uhpT* (MP1738), *pgtP* (MP1739), and *ushA2* (MP1779) *Salmonella* strains. The indicated strains were grown on MOPS-glucose-noble agar media containing 1 mM of (A) 2-aminoethylphosphonate (2-AP), (B) *sn*-glycerol-3-phosphate (Gly-3P), (C) glucose 6-P (G6-P, top) or fructose 6-P (F6-P, bottom), (D) phosphoenolpyruvate (PEP, top) or 3-phosphoglyceric acid (PGA, bottom), (E) ATP (top) or GTP (bottom). Diagrams under the plates outline the genomic context and expression profile of gene(s) being assayed. The bent arrows represent previously mapped transcriptional start sites (20, 28). Protein-coding sequences are depicted as large arrows that are colored according to their fold-change expression in wild-type cells subjected to P starvation (Fig. 2; Tables S1 and S2). (F) Positive and negative control plates for strains tested in (A–E). Plates contain either 1 mM Pi or no P source. Plates were incubated at 37°C during 16–18 h before being imaged. Images are representative of three independent experiments. Dashed lines separate non-contiguous sections of the same plate. Note instances of residual growth due to contaminating P source(s) in the agar and, in some cases, organic-P source.

Our RNA-seq data indicated that PhoB promotes the transcription of *phnSTUV*, *phnWX*, and *ugpBAEC* operons in response to P deprivation (Fig. 2; Table S2). The *phnSTUV* and *phnWX* operons encode components of a PhoB-dependent phosphonatase catabolic pathway, which mediates the uptake and degradation of 2-aminoethylphosphonate (2-AP) (1, 43 – 45). We confirmed that wild-type bacteria grow on 2-AP as the sole P source—an ability that is impaired by deletions of either *phnWRSTUV* or *phoB* (Fig. 4A). Notably, it has been reported that *Salmonella* can also utilize phosphonoacetic acid (PAA) as sole P source (46). However, contrary to this finding, we determined that both wild-type or *phoB* mutant *Salmonella* strains lack this ability (Fig. S4A through C). In fact, PAA degradation requires the relaxed specificity of phosphonatases encoded in the C-P lyase catabolic pathway. While this pathway is not present in *Salmonella*, it is found in several closely related species such as *Klebsiella aerogenes* (1, 45, 47), which can grow on PAA as the sole P source (Fig. S4C).

In *E. coli*, the *ugpBAECQ* locus encodes a PhoB-activated *sn*-glycerol-3-P (Gly-3P) ABC transporter (UgpBAEC) and a cytoplasmic phosphodiesterase capable of releasing Pi from this substrate (UgpQ). Together, these proteins enable *E. coli* to use Gly-3P as the sole P source (48 – 50). Similarly, we determined that *Salmonella* can grow on Gly-3P as the sole P source and that growth on this substrate is impaired by deletions of either *ugpBAEC* or *phoB* (Fig. 4B). Collectively, these results indicate that during P starvation, PhoB promotes the transcription of *phnSTUV*, *phnWX*, and *ugpBAEC* operons, thereby allowing *Salmonella* to scavenge P from 2-AP and Gly-3P, respectively.

### PhoB-independent genes support the growth of *Salmonella* on a variety of organic-Pi sources

We wondered if *Salmonella* could utilize additional P sources in a PhoB-independent manner. This bacterium harbors homologs of three major facilitator antiporters that can uptake a cognate negatively charged phosphate ester molecule in counterflow with a Pi anion (51 – 53). GlpT transports Gly-3P (54 – 56), UhpT translocates hexoses-6-phosphates (57), and PgtP uptakes 2- or 3-phosphoglyceric acid (PGA) and phosphoenolpyruvate (PEP) (58, 59). It has been suggested that these antiporters can promote a net Pi uptake by asymmetric self-exchange, where two external organic-Pi molecules are exchanged against one internal organic-Pi molecule, and that other cytoplasmic anions such as sulfate can participate in the exchange reaction (1, 52). We, therefore, tested the abilities of wild-type and *glpT*, *uhpT*, and *pgtP* mutant strains to grow on these cognate organic substrates as the sole P source.

We determined that GlpT was dispensable for growth on Gly-3P (Fig. S5A), which depends primarily on proteins encoded in the *ugpBAECQ* operon (Fig. 4B). In contrast, UhpT was required for the growth of *Salmonella* on medium containing either glucose-6-phosphate (G6-P) or fructose-6-phosphate (F6-P) as the sole P source. That is, a *uhpT* deletion hindered growth on G6-P or F6-P (Fig. 4C). Similarly, a *pgtP* deletion impaired growth on either PGA or PEP (Fig. 4D), indicating that PgtP allows *Salmonella* to utilize these substrates as the sole P source (59). Importantly, deletion of *phoB* did not affect the transcription of *uhpT* or *pgtP* (Fig. 2; Table S1), nor did it hinder the ability of *Salmonella* to grow on G6-P, F6-P, PGA, or PEP as the sole P source (Fig. 4C, D and F).

P acquisition from organic sources can also be attained by the activity of periplasmic enzymes that release Pi from organophosphates substrates to enable its uptake by Pi transporters such as PitA. *Salmonella* encodes three UDP-sugar/5′-nucleotidases paralogs (*ushA*, *ushA2*, and *ushA3*) as well as a periplasmic acid phosphatase (*aphA*). Whereas UDP-sugar/5′-nucleotidases can release terminal Pi from nucleoside mono-, di-, or triphosphates (60, 61), AphA has a relaxed substrate specificity being able to act on a wide range of organic substrates including some nucleotides (62, 63). Transcription of these genes was not affected by PhoB during P starvation, suggesting that these proteins are expressed in a PhoB-independent manner (Fig. 2; Table S1). Concordantly, we determined that wild-type and *phoB* mutant strains of *Salmonella* grew robustly on plates containing either AMP, ADP, ATP, or GTP as the sole P source (Fig. 4E; Fig. S5B and C). Whereas growth on these substrates was impaired by deletion of *ushA2*, inactivation of either *ushA*, *ushA3* or *aphA* did not hinder growth (Fig. 4E; Fig. S5B and C). Taken together, these results establish that the PhoB-independent gene products encoded by *uhpT*, *pgtP*, and *ushA2* allow *Salmonella* to efficiently use G6-P/F6-P, PGA/PEP, and AMP/ADP/ATP/GTP as sole P sources, respectively.

## DISCUSSION

In the current study, we define PhoB-dependent and independent transcriptional changes elicited by *Salmonella* in response to P starvation, inferring the identity of transcripts that may be under direct PhoB control. We confirm that PhoB promotes the transcription of genes required for the importation and utilization of 2-AP and Gly-3P as the sole P sources (43, 44). Contrary to a previous report (46), we establish that *Salmonella* is unable to utilize PAA as the sole P source. Finally, we show that *Salmonella* can utilize hexoses-6-phosphate (G6-P and F6-P), PGA/PEP, and nucleotides (ATP, ADP, AMP, and GTP) as their sole P source, and that the utilization of these compounds is PhoB independent.

The physiological response to P starvation has often been studied in an experimental context where bacteria are starved for P after being propagated in media containing excessive Pi. In this framework, cells experience a decrease in both extra-cytoplasmic and cytoplasmic Pi, which precipitates a molecular response aimed at restoring levels of cytoplasmic Pi (2, 6, 56, 64). In *Salmonella*, this response involves two broad patterns of transcriptional reprogramming (Fig. 1 and 2; Fig. S2 and Table S1). On the one hand, the lack of P promotes the PhoB-independent repression of several transcripts from genes that are associated with bacterial growth and replication (Fig. 1; Fig. S2 and Table S1). Most of these genes encode proteins involved in translation, including ribosomal proteins, elongation and release factors, and enzymes required for the biosynthesis of nucleotides and polyamines (65 – 68). Because nucleotides and rRNA are the largest intracellular reservoirs of assimilated P (2, 14, 69), inhibiting the transcription of these genes may prevent wasteful allocation of resources toward cellular components that require large quantities of P. Complementarily, the degradation of existing ribosomes induced by P starvation (70, 71) may replenish the pools of cytoplasmic nucleotides that are needed to sustain a transcriptional response to this stress.

On the other hand, P deficiency stimulates the transcription of a substantial number of PhoB-dependent genes (Fig. 1 to 3; Tables S1 and S2). Whereas these genes are primarily involved in the scavenging of environmental P and the retrieval of assimilated P from cellular components (1, 6, 40, 49, 54, 72), they may also help cells to cope with secondary stresses. For instance, P starvation will lead to growth arrest (Fig. S1B). However, under aerobic conditions, cells will continue to import and metabolize carbon, thereby generating endogenous reactive oxygen species, which are no longer diluted by growth and may damage cellular components (73 – 77). In *Salmonella*, PhoB promotes the transcription of the catalases encoded by the *katE* and *katN* genes, as well as a dedicated cytochrome bd terminal oxidase encoded by the *cydB* gene (located within the STM14_*RS02410-cydA-cydB-RS02415* operon). Whereas CydB may reduce endogenous hydrogen peroxide generation by improving oxygen consumption (76), the catalases detoxify any hydrogen peroxide that may be produced from oxygen that escapes the respiratory chain.

Here, we show that *Salmonella* uses *uhpT*, *pgtP*, and *ushA2* gene products to grow on G6-P/F6-P, PGA/PEP, and ATP/ADP/AMP/GTP as the sole P source, respectively (Fig. 4C through E; Fig. S5). Notably, under our experimental conditions, the transcription of these genes is independent of PhoB and the availability of P in the growth medium (Fig. 2; Table S1). Furthermore, because a mutation in *phoB* does not impact growth on these substrates as the sole P source (Fig. 4C through E), PhoB does not control UhpT, PgtP, nor UshA2 expression or activity. Interestingly, *E. coli* harbors a UhpT homolog that shares 100% amino acid sequence identity with the UhpT protein from *Salmonella*. In *E. coli*, it was reported that PhoB represses UhpT expression, and that this bacterium is unable to utilize G6-P as the sole P source (78). While we have not measured the effect of PhoB on *uhpT* transcription in *E. coli*, we have found that this organism is able to grow on G6-P or F6-P, two UhpT substrates, as the sole P source (Fig. S6).

In *Salmonella*, transcription of the nucleotidase *ushA2* remains constant over a wide range of growth conditions (19, 20). Transcription of *uhpT* and *pgtP*, on the other hand, is controlled by dedicated two-component signal transduction systems that are activated by their cognate sugar-phosphate substrates (79 – 82). For *uhpT*, transcription initiation is also stimulated by cyclic AMP receptor protein (Crp)-cyclic AMP (Crp-cAMP) (83, 84), indicating that hexoses-6-phosphates are typically acquired in the context of carbon, rather than P starvation. That UhpT and PgtP can supply P to the cytoplasm, allowing bacterial growth in the absence of environmental Pi in a PhoB-independent manner, suggests that these proteins may be exploited to understand how PhoB/PhoR senses P limitation to initiate signal transduction (85).

## MATERIALS AND METHODS

### Bacterial strains, plasmids, oligonucleotides, and strain construction

The bacterial strains and plasmids used in this study are listed in Table S5 and oligonucleotide sequences are presented in Table S6. All *Salmonella enterica* serovar Typhimurium strains are derived from strain 14028s (86) and were constructed by lambda Red-mediated recombination (87). Deletions and gene fusions generated via this method were subsequently moved into clean genetic backgrounds via phage P22-mediated transduction as described (88). Bacterial strains used in recombination and transduction experiments were grown in Luria–Bertani (LB) medium at 30°C or 37°C (87, 88). When required, the LB medium was supplemented with ampicillin (100 µg/mL), chloramphenicol (20 µg/mL), kanamycin (50 µg/mL), gentamicin (18 µg/mL), apramycin (80 µg/mL), or tetracycline (20 µg/mL).

### Growth conditions

Physiological growth experiments either on solid or liquid media were carried out at 37°C in MOPS minimal medium (89) supplemented with 22 mM glucose, 5 mM MgSO4, an amino acids mixture (1.6 mM of alanine, glycine, leucine, glutamate, and serine; 1.2 mM glutamine, isoleucine, and valine; 0.8 mM arginine, asparagine, aspartate, lysine, phenylalanine, proline, threonine, and methionine; 0.4 mM histidine and tyrosine, and 0.2 mM cysteine and tryptophan), and the indicated P source. The following organic-P compounds were used at 1 mM: 2-aminoethylphosphonic acid, adenosine 5′-monophosphate disodium salt, adenosine 5′-diphosphate disodium salt dihydrate, adenosine 5′-triphosphate disodium salt, guanosine 5′-triphosphate sodium salt, fructose-6-phosphate disodium salt, glucose-6-phosphate disodium salt, phosphoenol pyruvic acid monopotassium salt, phosphonoacetic acid, and 3-phosphate bis(cyclohexylammonium) salt. All P sources were purchased from Sigma-Aldrich.

For experiments on solid media, 1.5% (wt/vol) noble agar (Difco) was added into MOPS minimal medium described above and supplemented with 1 mM of the indicated P source. For assays on liquid media, experiments were conducted as follows: after overnight (∼16 to 20 h) growth in MOPS medium containing 1 mM K_2_HPO_4_, cells were inoculated (1:100) in a similar fresh MOPS medium and grown until an optical density at 600 nm (OD_600_) of approximately 0.4. At that timepoint, cells were either Pi-downshifted or kept in Pi-rich (1 mM) media as control.

### RNA isolation and sequencing

Four independent biological replicates of wild-type and *phoB* mutant strains were grown in MOPS medium supplemented with 1 mM K_2_HPO_4_ to an OD_600_ of 0.4–0.45. Bacterial cultures were subsequently split into two equal fractions of approximately 10 OD_600_ units, and were labeled either as “−P” or “+Pi”. −P-treatment cells were washed twice (6,000 × *g*, 5 min) with MOPS medium lacking K_2_HPO_4_ (or any other P source). Conversely, +Pi-treatment cells were washed in MOPS medium supplemented with 1 mM K_2_HPO_4_. Bacterial cultures were then grown for 35 min to induce the Pi-starvation response. Cultures were then treated with RNAprotect (Qiagen) for 5 min at room temperature to stabilize mRNA, and then collected by centrifugation (6,000 × *g*, 5 min). Total RNA was isolated by hot-phenol extraction and further purified using the RNeasy Kit (Qiagen) with on-column DNase I treatment. The eluates were treated with Turbo DNase (Invitrogen) for 30 min before a second round of purification using the RNeasy Kit. rRNA was depleted using the NEBNext rRNA Depletion kit (Bacteria) and purified from the enzymatic reactions using RNAClean XP magnetic beads (Beckman Coulter) following the manufacturer’s instructions. RNA quality and ribosomal depletion were assessed using BioAnalyzer (Agilent). RNA-seq was performed at the Genomics Core Facility (Penn State University). Sequencing mRNA libraries were prepared using Illumina Tru-seq Stranded mRNA library kit. Approximately 5 million 75-nt single reads per sample were determined using a NextSeq High Output (Illumina) with >96% of the reads having a Q30 quality.

### RNA-seq mapping and differential gene expression analyses

Untrimmed reads were aligned to the *Salmonella enterica* serovar Typhimurium 14028s reference genome (NC_016855.1; NC_016856.1) using the SeqMan NGen (DNASTAR v17.0.2.1). Assembly files were uploaded into Geneious Prime v11 software (90). Reads Per Kilobase of transcript, per Million mapped reads (RPKM), Fragments Per Kilobase of transcript per Million mapped reads (FPKM), and Transcripts Per Million (TPM) read counts were calculated using Geneious prior to differential expression analysis using the DeSeq2 Geneious plugin (91) with a false discovery rate of 0.1. Genes with log_2_ fold changes above and adjusted *P*-values less than 0.01 were considered significantly different. Tables S1 and S2 contain gene lists, adjusted *P*-values, and log_2_ fold changes for transcriptome comparisons.

### Immunoblot analysis

*cydB-* and *phoN2*-HA-tagged *Salmonella* cells were downshifted to P-lacking MOPS medium for 35 min. Alternatively, for the detection of ApeE-HA, ZitB-HA, and CydB-HA, cells were grown in MOPS medium with either 50 µM or 1 mM K_2_HPO4 during 18 h. Equivalent amounts of bacterial cells normalized by OD_600_ values were collected, washed with PBS, and suspended in 50 mM Tris-HCl pH 8.0, 0.5% SDS sample buffer, and 1× protease inhibitor (Roche). Cells were lysed in a Mini-Beadbeater-96 (BioSpec) and insoluble debris was removed by centrifugation (10 min, 10,000 × *g*, 4°C). Protein extracts were quantified using a Rapid Gold BCA Protein Assay Kit (Pierce), mixed with 4× Laemmli sample buffer, and boiled for 5 min. Equal amounts of protein samples within each group were loaded and resolved using 4%–12% NuPAGE gels (Life Technologies), and subsequently electro-transferred onto nitrocellulose membrane (iBlot; Life Technologies) following the manufacturer’s protocol. C-terminal HA-tags were detected using Direct-Blot HRP anti-DYKDDDDK Tag Antibody (BioLegend). The blots were developed with the SuperSignal West Femto Chemiluminescent system (Pierce) and visualized with an Amersham Imager 600 (GE Healthcare Life Sciences). Mouse anti-RpoB antibody (Thermo Fisher Scientific) was used as the loading control.

### Image acquisition, analysis, and manipulation

Plate images were acquired using an Amersham Imager 600 (GE Healthcare Life Sciences). ImageJ software (92) was used to crop the edges, rotate, and adjust the brightness and contrast of the images. These modifications were simultaneously performed across the entire set of images to be shown. Noncontiguous sectors from single plates are indicated by dashed lines.

## Data Availability

The RNA-seq data set is available as GEO submission GSE227715.

## SUPPLEMENTAL MATERIAL

Fig. S1 spectrum.02260-23-s0001.tif (A) Fluorescence from wild-type (14028s) and *phoB* mutant (EG9054) strains of *Salmonella* carrying pP*phoB-gfp*_AAV_ or pP*pstS-gfp*_AAV_. (B) Growth curve of wild-type (14028s) or *phoB* (EG9054) *Salmonella* harboring pP*pstS-gfp*_AAV_. (C) Viable cell counts of wild-type (14028s) and *phoB* (EG9054) *Salmonella* at the beginning and end of Pi downshift.

Fig. S2 spectrum.02260-23-s0002.tif
*Salmonella* PhoB-independent response to P starvation.

Fig. S3 spectrum.02260-23-s0003.tif
*Salmonella* PhoB binding motif.

Fig. S4 spectrum.02260-23-s0004.tif Utilization of phosphonoacetic acid as sole P source.

Fig. S5 spectrum.02260-23-s0005.tif
*Salmonella* genetic requirements for the utilization of glycerol-3-phosphate and nucleotides as the only P source.

Fig. S6 spectrum.02260-23-s0006.tif Utilization of G6-P and F6-P by *E. coli*.

Supplemental Material Text spectrum.02260-23-s0007.docx Tables S3 to S6, sequences of PhoB-activated genes containing putative PhoB-binding sites, and legends for Fig. S1 to S6.

Table S1 spectrum.02260-23-s0008.xls RNA-Seq summary.

Table S2 spectrum.02260-23-s0009.xls PhoB activated genes.

ASM does not own the copyrights to Supplemental Material that may be linked to, or accessed through, an article. The authors have granted ASM a non-exclusive, world-wide license to publish the Supplemental Material files. Please contact the corresponding author directly for reuse.
