# Supplementary figures and images for: Phosphorus starvation response and PhoB-independent utilization of organic phosphate sources by Salmonella enterica
Source: Microbiol Spectr. 2023 Oct 3;11(6):e02260-23. doi: 10.1128/spectrum.02260-23 (PMC10715179; doi:10.1128/spectrum.02260-23)

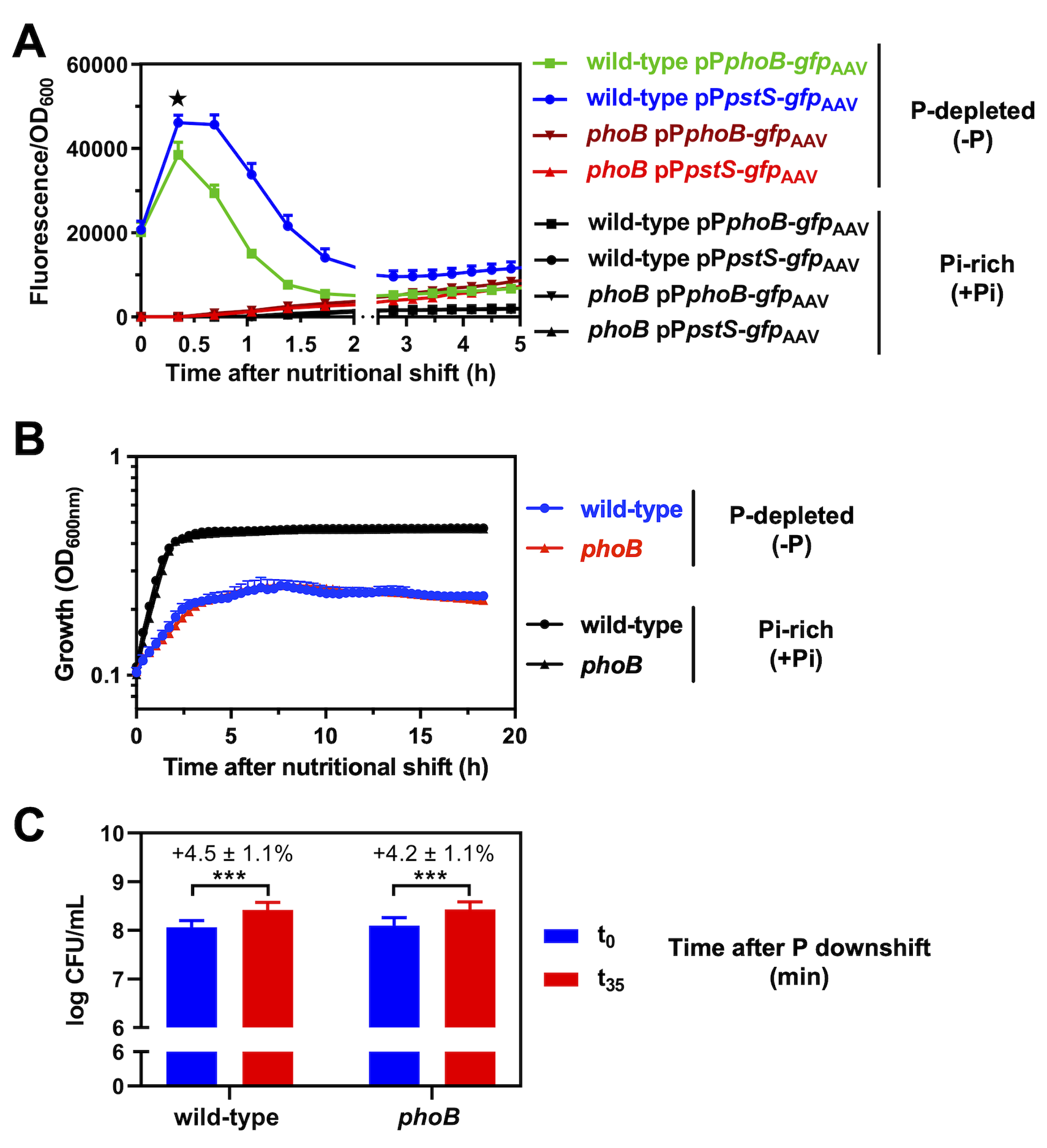

Supplement: Fig. S1 — (A) Fluorescence from wild-type (14028s) and phoB mutant (EG9054) strains of Salmonella carrying pPphoB-gfp AAV or pPpstS-gfp AAV. (B) Growth curve of wild-type (14028s) or phoB (EG9054) Salmonella harboring pPpstS-gfp AAV. (C) Viable cell counts of wild-type (14028s) and phoB (EG9054) Salmonella at the beginning and end of Pi downshift. [file spectrum.02260-23-s0001.tif]

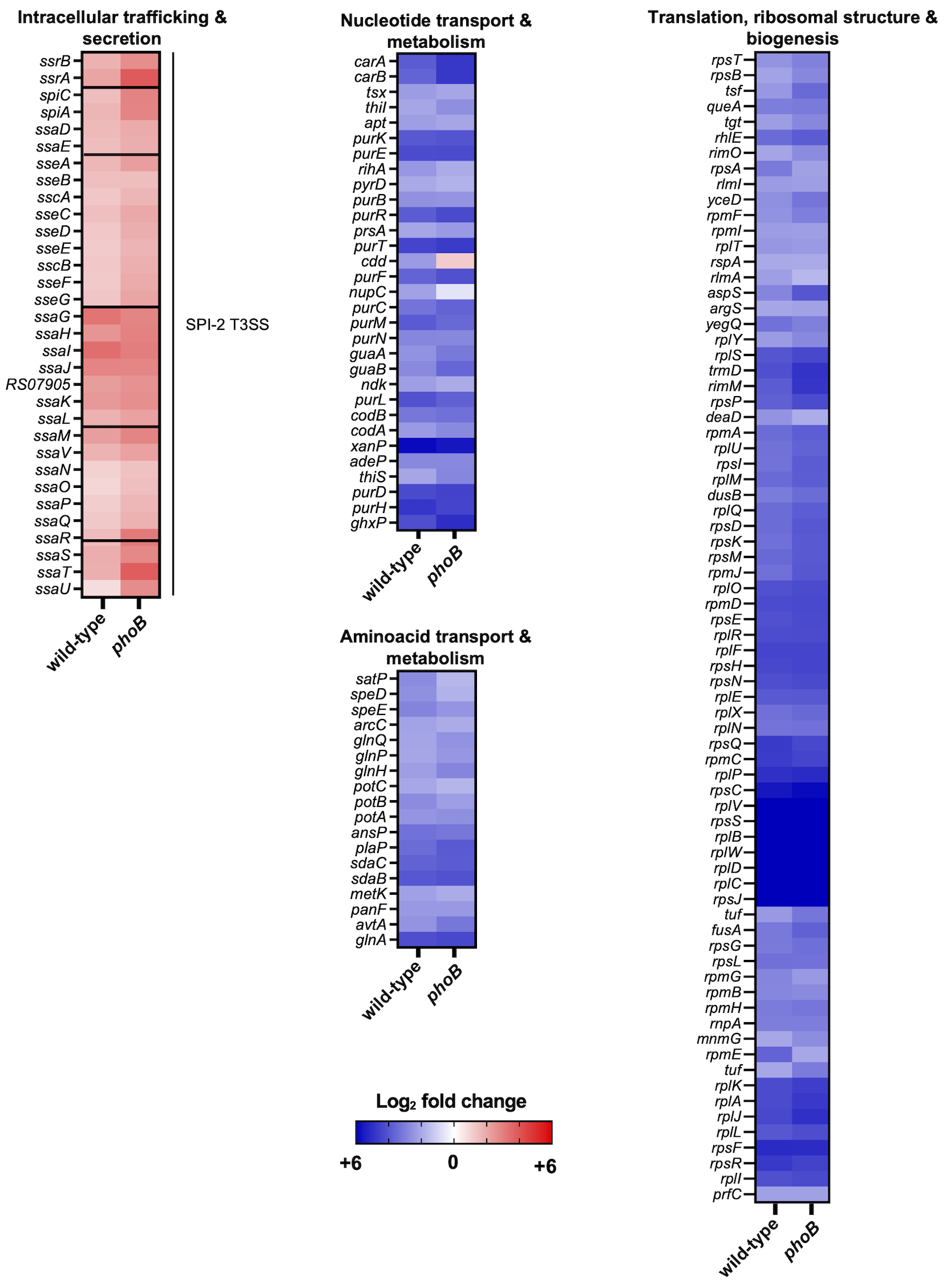

Supplement: Fig. S2 — Salmonella PhoB-independent response to P starvation. [file spectrum.02260-23-s0002.tif]

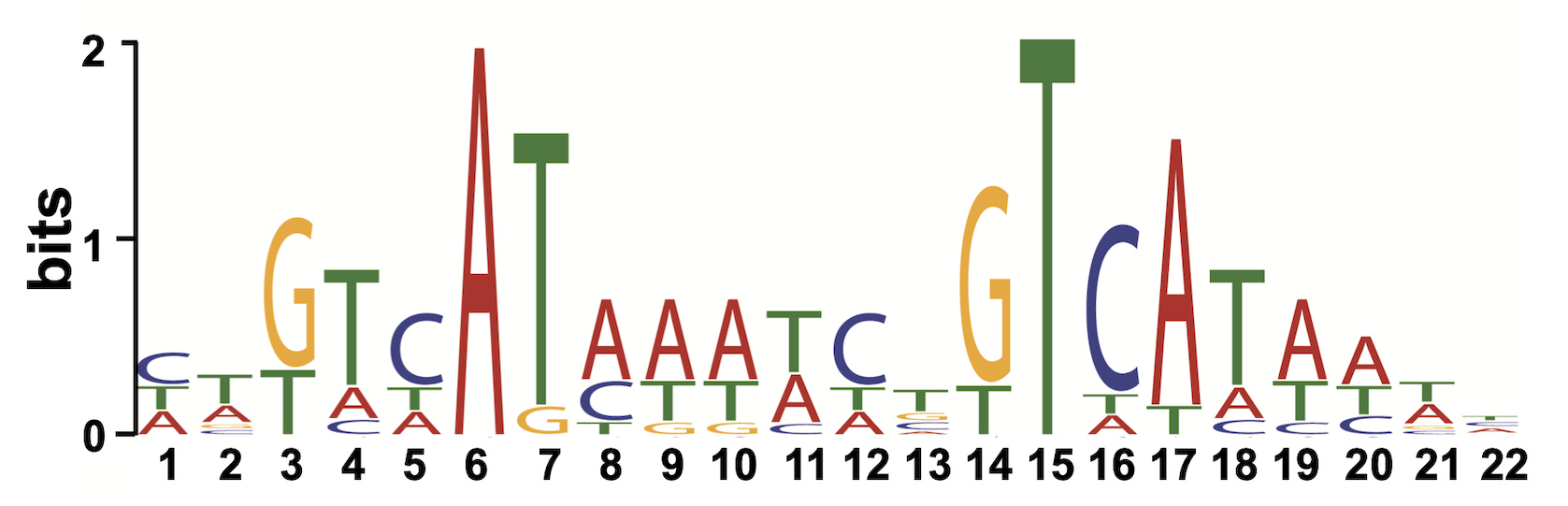

Supplement: Fig. S3 — Salmonella PhoB binding motif. [file spectrum.02260-23-s0003.tif]

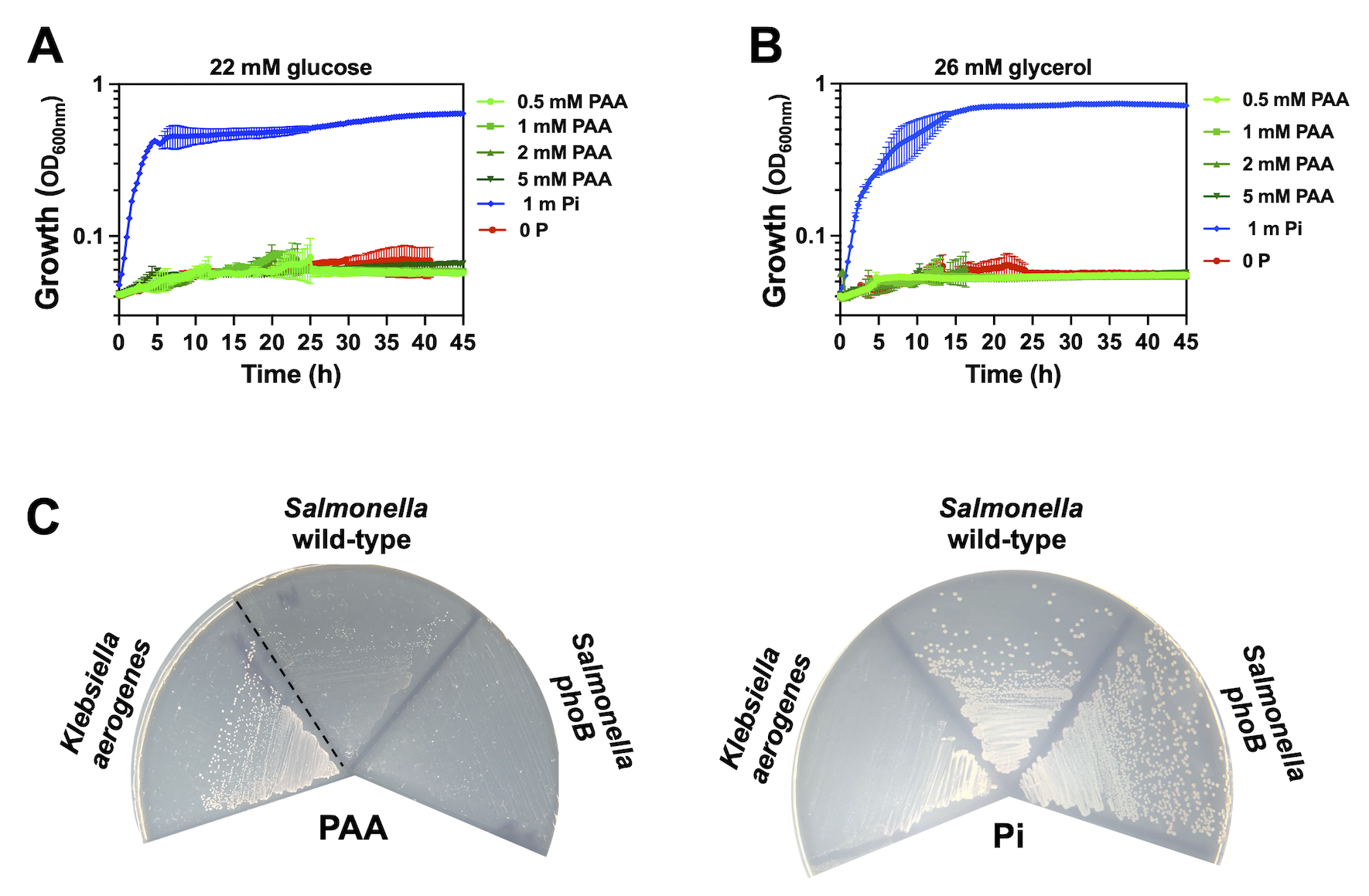

Supplement: Fig. S4 — Utilization of phosphonoacetic acid as sole P source. [file spectrum.02260-23-s0004.tif]

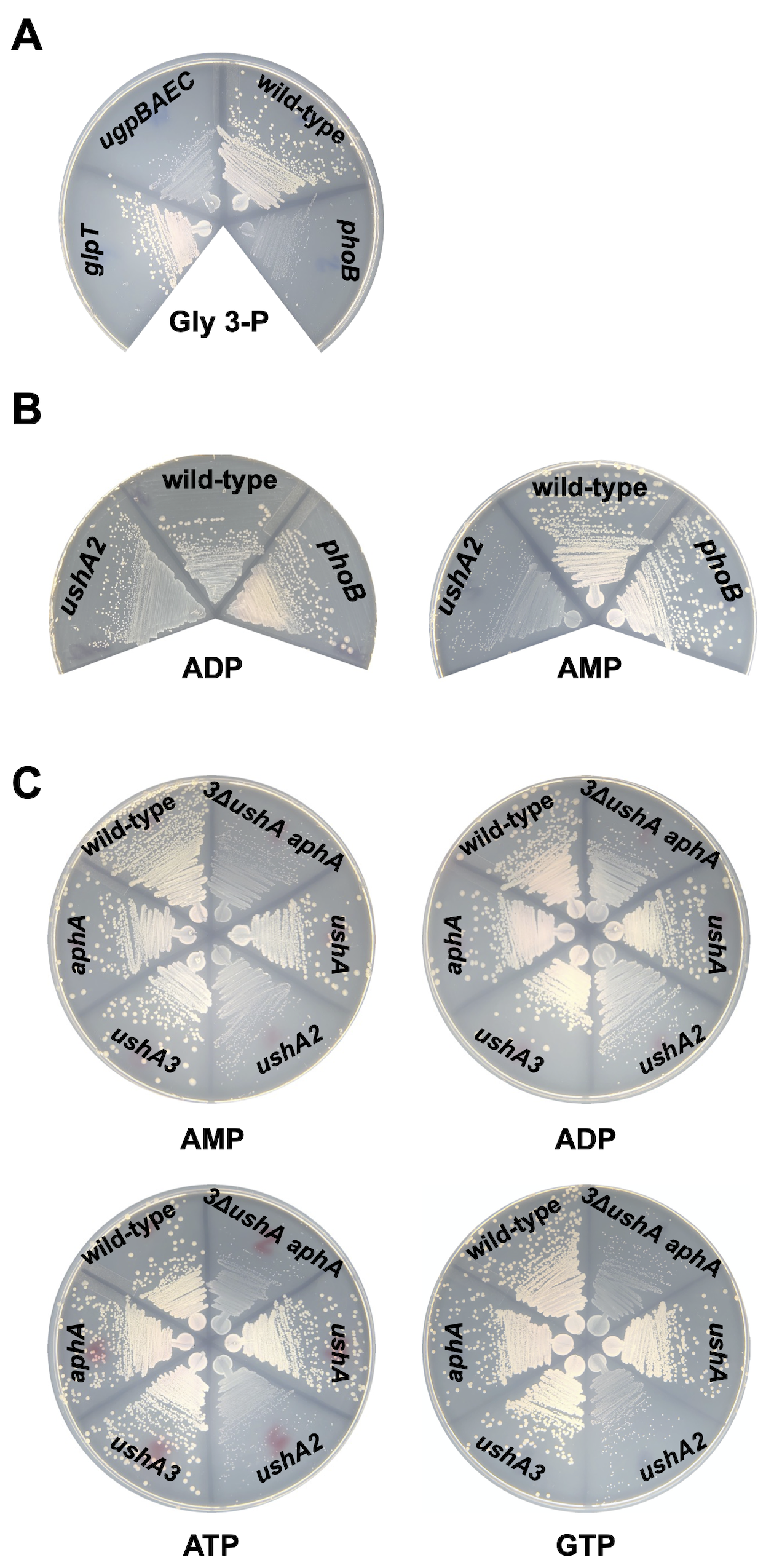

Supplement: Fig. S5 — Salmonella genetic requirements for the utilization of glycerol-3-phosphate and nucleotides as the only P source. [file spectrum.02260-23-s0005.tif]

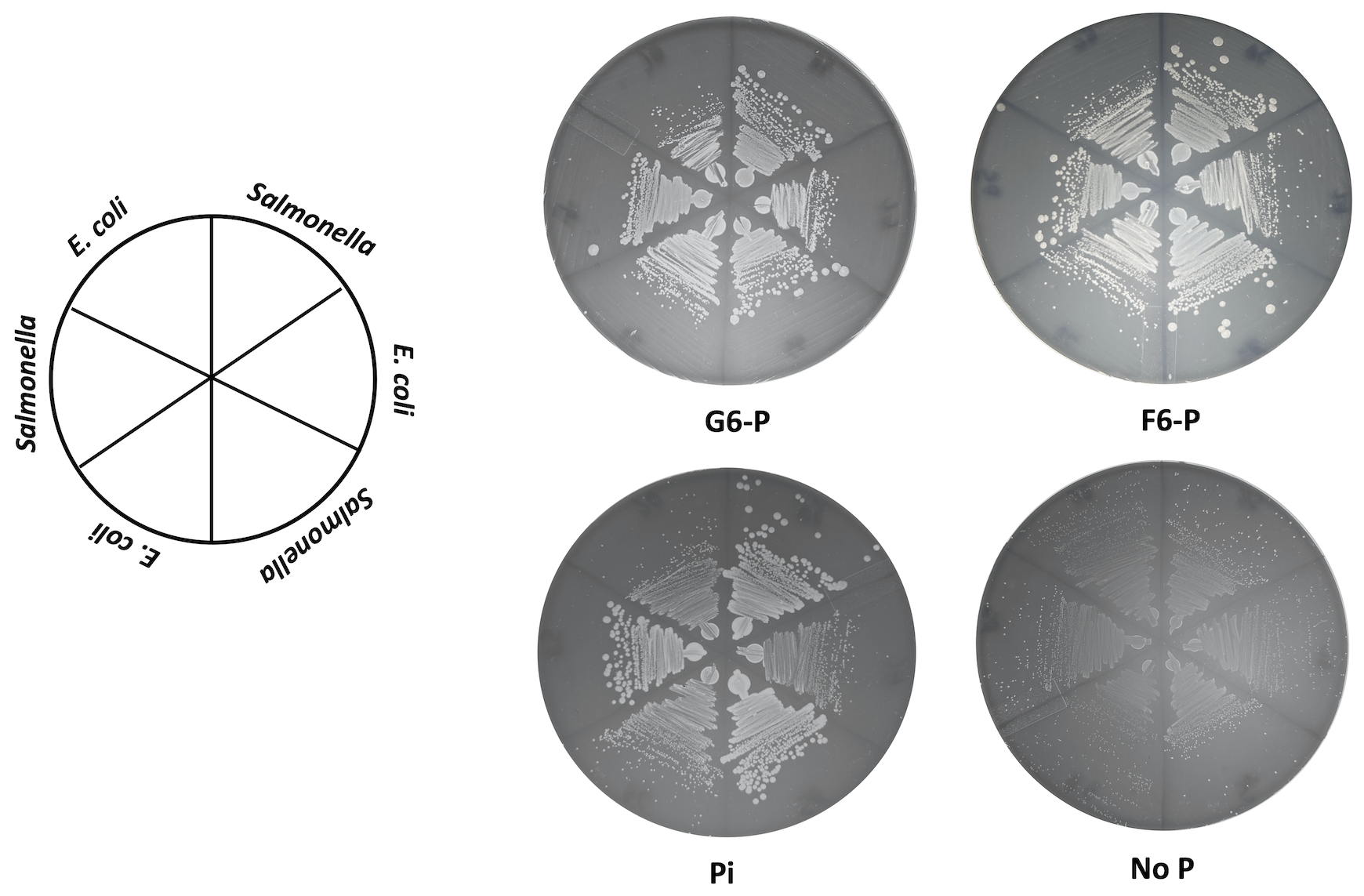

Supplement: Fig. S6 — Utilization of G6-P and F6-P by E. coli. [file spectrum.02260-23-s0006.tif]
