# Supplementary material for: Phosphorus starvation response and PhoB-independent utilization of organic phosphate sources by Salmonella enterica
Source: Microbiol Spectr. 2023 Oct 3;11(6):e02260-23. doi: 10.1128/spectrum.02260-23 (PMC10715179; doi:10.1128/spectrum.02260-23)
Supplement: Supplemental Material Text — Tables S3 to S6, sequences of PhoB-activated genes containing putative PhoB-binding sites, and legends for Fig. S1 to S6. [file spectrum.02260-23-s0007.docx]

**Supplemental Material**

**Table S1. RNA-Seq summary.**

**Table S2. PhoB activated genes.**

**Table S3. Canonical PhoB-regulated genes used as input for MEME software.**

| **Gene name** | **Strand** | **Sequence motif identified by MEME** | **Distance (bp) to translation start site** | **p-value** |
| --- | --- | --- | --- | --- |
| *phoB* | + | **TTTTCAT**AAAT**CTGTCAT**AAAT | -50 | N/A |
| *pstS* | +  + | **TTGTCAT**CAAA**CCGTCAT**AATC  **AAGACAT**ATAA**CTGTCAT**CAAT | -86  -64 | N/A |
| *phoE* | -  + | **CGGTAAT**AAAA**TGGTCAA**ATTC  **TTGTCAT**AAAT**CTTTCAT**TACC | -133  -74 | N/A |
| *ugpB* | + | **AAGTTAT**TTTT**CTGTCAT**TCGA | -72 | N/A |
| *phnS* | + | **CTTTCAT**CGTT**TTGTCAT**ATAA | -68 | N/A |
| *psiE* | **+** | **AATATAG**ATGC**CCGTCAC**ATTT | -44 | N/A |
| *ytfK* | **+** | **CTGTAAT**CAAA**AGGTAAA**TATA | -121 | N/A |
| *waaH* | **+** | **CTGTAAA**AATT**AATTATG**GCGG | -67 | N/A |

**Table S4. Putative PhoB-motifs predicted by FIMO.**

| **Gene name/NCBI locus tag** | **Strand** | **Sequence motif identified by FIMO** | **Distance (bp) to translation start site** | **p-value** |
| --- | --- | --- | --- | --- |
| *yeaG* | - | GTGATATTAAACGGTTACATTG | -341.5 | p<0.001 |
|  | - | CGGTCACATAACGATCATCAGC | -293.5 | p<0.001 |
|  | + | TATTCATATGAACGGCTCTTAA | -42.5 | p<0.001 |
| *yncC* | - | CCGTTTTATCTCCGTCATTCCT | -182.5 | p<0.001 |
| *yiaG* | + | TTGTTATTTTATTGTTAATTTT | -281.5 | p<0.0001 |
|  | + | GTGTAATATTTTTTTAATCTTT | -209.5 | p<0.001 |
| *phnO* | **+** | CCGCCGTTTAACTGTCATAGAA | -48.5 | p<0.001 |
|  | - | CCGTCATTATTTATTCTATGAC | -35.5 | p<0.001 |
| *zitB* | - | CCGTTATCAGACTTTTTTTTGC | -98.5 | p < 0.0001 |
|  | - | TAGACATCATACTGATTTTTCG | -44.5 | p < 0.001 |
| *katN* (*STM14_RS09555*) | - | CAGCCATTTGCATTTCTTCTTT | -184.5 | p < 0.001 |
| *treZ* | - | CTTCCATCTGTCCGTTTTTATC | -325.5 | p < 0.0001 |
|  | + | TTATCAGCGAACGTTTATTTAA | -207.5 | p < 0.001 |
| *fbaB* | + | ATTTAATAATACCTTTTAAATA | -199.5 | p < 0.0001 |
|  | - | AAGTGTTAGATCGGTCAAAATT | -165.5 | p < 0.001 |
| *talA* | - | AGGTAACATGACCGTTATAAGT | -130.5 | p < 0.001 |
|  | - | CCGTTATAAGTTGATAACAAAC | -141.5 | p < 0.001 |
| *cstA* | + | ATGTAAAAAATGGGTAACAATC | -105.5 | p < 0.001 |
| *ybdD* | - | TTGTCACCTGTCATTCAAATGC | -13.5 | p < 0.001 |
| *STM14_RS03615* | - | TAGACATCTAAACGTCTTGATT | -53.5 | p < 0.0001 |
| *ybdR* | + | CTGCTATTTGTCTGGCAATTTT | -152.5 | p < 0.001 |
|  | + | TGTTAATCAATACTGAATTTAT | -198.5 | p < 0.001 |
| *wrbA* | + | AAGAAATAAATAAGTTATTCTT | -188.5 | p < 0.0001 |
|  | + | AATAAATAAGTTATTCTTATAT | -184.5 | p < 0.0001 |
|  | + | CCGTCATAGATTAGACATCATA | -364.5 | p < 0.001 |
| *narK* | + | AAGTTACAAATAATTTAATAAA | -204.5 | p < 0.001 |
|  | + | ATTCTAAAAAAACTTCAATAAG | -86.5 | p < 0.001 |
| *STM14_RS12065* | - | CCGCAATAATACCGGCATTAAA | -204.5 | p < 0.0001 |
|  | + | CGGTTATCATTCCTGATTTTTC | -70.5 | p < 0.001 |
| *STM14_RS15000* | + | TGGGCATTTATCCTTAATATCC | -175.5 | p < 0.001 |
|  | + | CTGACATTACTCGGATATATTC | -151.5 | p < 0.001 |
| *apeE* | + | TTTTAACCAGAATGTTTAAAAC | -82.5 | p < 0.001 |
|  | - | ATGCCATTCAGCAGTCATCATA | -274.5 | p < 0.001 |
| *yciG* | - | TAGAGATAATATATTCTATATT | -154.5 | p < 0.001 |
| *yciE* | + | TCGAAATAATATTTTTAATTAT | -71.5 | p < 0.0001 |
|  | - | TTTAAATCAGTTTGTTTTTCCT | -142.5 | p < 0.001 |
| *bapA* | - | TATTTATCTTTATGTTTTATAA | -338.5 | p < 0.0001 |
|  | - | TTTTAATTTTTTCTTCTTTTTC | -364.5 | p < 0.0001 |
|  | + | CCTTTATCAATAGATCTTAATT | -241.5 | p < 0.001 |
|  | - | CAGCAATATTAAATTTTATAAA | -424.5 | p < 0.001 |
|  | + | ACTTCAAATGATTTTTATAAAA | -437.5 | p < 0.001 |
|  | - | TAGTCAGAAAAAAATCTTTCTG | -217.5 | p < 0.001 |
| *phoN2* | + | GTATCATCAAACCGTCAACTGC | -81.5 | p < 0.001 |
| *psiE* | - | TGTTTATATTTTGTTCAATAAG | -86.5 | p < 0.0001 |
|  | + | AATATAGATGCCCGTCACATTT | -55.5 | p < 0.0001 |
|  | - | CGGGCATCTATATTTTTTATTT | -63.5 | p < 0.001 |
| *STM14_RS04420* | - | AATCTTGCAATAATTCATAAAC | -127.5 | p < 0.001 |
| *STM14_RS08420* | - | TTTACATAATACCATCACATGG | -125.5 | p < 0.001 |
|  | - | CGGTAATAAATCTTTGGCAATC | -183.5 | p < 0.001 |
|  | - | AAGATATATTTTTTGAAAACAT | -150.5 | p < 0.001 |
| *STM14_RS09875* | - | CGGACATAAAAATTTATTTATC | -91.5 | p < 0.0001 |
|  | + | TATACTTAAAACGTTTTTACTG | -27.5 | p < 0.001 |
|  | - | AATTTATTTATCGCTATTAAGT | -101.5 | p < 0.001 |
| *STM14_RS19935* | + | ACGATCTCAATACGTCTCATTA | -205.5 | p < 0.001 |
| *STM14_RS23690* | - | CATTTATATTAATATCATTAAT | -227.5 | p < 0.0001 |
|  | + | TATTAATATAAATGATATAAAA | -218.5 | p < 0.001 |
| *STM14_RS23900* | + | AGTTCAGTAATTCGTCGTAATT | -109.5 | p < 0.001 |

**Table S5. Bacterial strains and plasmids used in this study.**

| **Strain** | **Relevant characteristics** | **Identifier** | **Source** |
| --- | --- | --- | --- |
| ***Escherichia coli*** | | | |
| EC100D | *pir*^+^ (DHFR) host strain used for generation and propagation of plasmid constructs | EC100D | Epicentre |
| MG1655 | wild-type | MG1655 | 1 |
| ***Klebsiella aerogenes*** | | | |
| ATCC 13048 | wild-type | ATCC 13048 | ATCC |
| ***Salmonella enterica* serovar Typhimurium** | | | |
| 14028s | wild-type | 14028s | 2 |
| EG9054 | *phoB*::Km^R^ | *phoB* | 3 |
| RB437 | *zitB*-HA::Cm  (*STM14_RS04415*-HA::Cm^R^) | *zitB-*HA | This study |
| RB438 | *cydB*-HA::Cm  ( *STM14_RS02420*-HA::Cm^R^) | *cydB-*HA | This study |
| RB439 | *yciG*-HA::Cm  (*STM14_RS09540*-HA::Cm^R^) | *yciG-*HA | This study |
| RB440 | *apeE*-HA::Cm (*STM14_RS19030*-HA::Cm^R^) | *apeE-*HA | This study |
| RB441 | *phoN2*-HA::Cm  (*STM14_4324*-HA::Cm^R^) | *phoN2*-HA | This study |
| RB443 | *phoB*::Km^R^ *zitB*-HA::Cm^R^ | *phoB zitB-*HA | This study |
| RB444 | *phoB*::Km^R^ *cydB*-HA::Cm^R^ | *phoB cydB-*HA | This study |
| RB445 | *phoB*::Km^R^ *yciG*-HA::Cm^R^ | *phoB yciG-*HA | This study |
| RB446 | *phoB*::Km^R^ *apeE*-HA::Cm^R^ | *phoB apeE-*HA | This study |
| RB447 | *phoB*::Km^R^ *phoN2*-HA::Cm^R^ | *phoB phoN2-*HA | This study |
| MP1736 | *ΔugpBAEC*::Cm^R^ | *ugpBAEC* | This study |
| MP1737 | *ΔglpT::*Gm^R^ | *glpT* | This study |
| MP1738 | *ΔuhpT::*Ap^R^ | *uhpT* | This study |
| MP1739 | *ΔpgtP::*Tn*10* (Tet^R^) | *pgtP* | This study |
| MP1778 | *ΔushA*::Km^R^  (*STM14_* *RS00735*::Km^R^ ) | *ushA* | This study |
| MP1779 | *ΔushA2*::Gm^R^  (*STM14_RS03085*::Gm^R^ ) | *ushA2* | This study |
| MP1780 | *ΔushA3*::Tn*10*  (*STM14_* *RS21590* ::Tn*10* ) | *ushA3* | This study |
| MP1784 | *ΔphnWRSTUV*::Cm^R^ | *phnWRSTUV* | This study |
| MP1785 | *ΔaphA*::Cm^R^ | *aphA* | This study |
| MP1796 | *ΔushA*::Km^R^ *ΔushA2*::Gm^R^ *ΔushA3*::Tn*10* (Tet^R^)  *ΔaphA*::Cm^R^ | *ushA ushA2 ushA3 aphA; 3ΔushA aphA* | This study |
| **Plasmid** | **Relevant characteristics** | | **Source** |
| pSIM6 | rep_pSC101_^ts^ Amp^R^ P_CI857_-γβexo | | 4 |
| pKD3 | rep_R6Kγ_ Amp^R^ FRT Cm^R^ FRT | | 5 |
| pKD4 | rep_R6Kγ_ Amp^R^ FRT Km^R^ FRT | | 5 |
| pKD4-Ap^R^ | rep_R6Kγ_ Amp^R^ FRT Ap^R^ FRT | | 6 |
| pKD4-Gm^R^ | rep_R6Kγ_ Amp^R^ FRT Gm^R^ FRT | | 6 |
| pKD4-Tn*10* | rep_R6Kγ_ Amp^R^ FRT Tn*10* (Tet^R^) FRT | | 6 |
| pGFP_AAV_ | rep_pMB1_ Amp^R^ promoterless *gfp*_AAV_ vector control | | 3 |
| pP*phoB*-*gfp*_AAV_ | rep_pMB1_ Amp^R^ P*phoB*-*gfp*_AAV_ | | 3 |
| pP*pstS*-*gfp*_AAV_ | rep_pMB1_ Amp^R^ P*pstS*-*gfp*_AAV_ | | 3 |

**Table S6. Oligonucleotides sequences used in this study**

| **Name** | **Sequence (5’→ 3’)** | **Purpose** |
| --- | --- | --- |
| 801 | ACGCGTGATATCGCGCATC | *ugpBAEC*::Cm^R^ verification |
| 802 | CACAAAAAGAGAGATAACCGATGATATCGTTACGACATACAGCTTCATATGAATATCCTCCTTA | *ugpBAEC* inactivation |
| 803 | TGTTGCAGCAGGACAGCGGGACGCCGCAGCCTGACATCCCGCGTGTAGGCTGGAGCTGCTTC | *ugpBAEC* inactivation |
| 782 | AGCGCGCTTGCCAGCGGCG | *glpT*::Gm^R^ verification |
| 783 | CGCTGGCAGATTTTCCTGGGGATATTCTTTGGCTATGCCGGTGTAGGCTGGAGCTGCTTC | *glpT* inactivation |
| 784 | CGCTTGCAGCGACAGAACCGCCCAGGTAACCAAACAGACCATATGAATATCCTCCTTA | *glpT* inactivation |
| 779 | ACCAGGTGCGCAAGCCGAC | *uhpT*::Ap^R^ verification |
| 780 | GCGGCGTAAAATGTGGTTCAAGCCGTTCATGCAGTCCTGTGTAGGCTGGAGCTGCTTC | *uhpT* inactivation |
| 781 | ATAAGCGAAGGTGCCCTTGATACCGTCCGCAGCGCCGATGCATATGAATATCCTCCTTA | *uhpT* inactivation |
| 776 | AGGGCAATCGGCGCATAAA | *pgtP*::Tn*10* |
| 777 | CCGGAAAAAGTCCAGGCCACATATGGTCGATATCGTATACGTGTAGGCTGGAGCTGCTTC | *pgtP* inactivation |
| 778 | GCCAGCCTGCCAGTAGCGTGGAGGGAATCGCCGCCCACTCCATATGAATATCCTCCTTA | *pgtP* inactivation |
| 898 | ATTACCTGATCGGCTTCGA | *ushA*::Km^R^ *verification* |
| 899 | CGACTACAGGCATTGTTTTTCTTTAATGTAGCGTAAATGGGTGTAGGCTGGAGCTGCTTC | *ushA* *(STM14_RS00735)* inactivation |
| 900 | GTATGTTATGCGGCCGGTTATCGACCGCATAACATTATGGCCATATGAATATCCTCCTTA | *ushA* *(STM14_RS00735)* inactivation |
| 901 | ATGCGATGTTGGAGATAGT | *ushA2*::Gm^R^ verification |
| 902 | AGGTAATTTCTGCGGTTGATATTGAGTCAGGGAGAGAAAGGTGTAGGCTGGAGCTGCTTC | *ushA2* (*STM14_RS03085*) inactivation |
| 903 | TAAGGTTGCGCGCCATCAGGCAGAAATGGCTATCCGTACCCATATGAATATCCTCCTTA | *ushA2* (*STM14_RS03085)* inactivation |
| 904 | GCAGGCATTAATGGTGAACA | *ushA3*::Tn*10* verification |
| 905 | TAACGTAGCGAATCTTTATATGACTGAAAGGGACTTATTTGTGTAGGCTGGAGCTGCTTC | *ushA3* (*STM14_RS21590*) inactivation |
| 906 | CATGTCACGCCGCGACACTGAACGCGCCGCGGCAGGGGAAACATATGAATATCCTCCTTA | *ushA3* (*STM14_RS21590*) inactivation |
| 895 | GCGTTATGGTCAGATAGT | *aphA*::Cm^R^ verification |
| 896 | ATCTTAATAATTATAATATTTTGAATTTTAAGGGAAAACCCATATGAATATCCTCCTTA | *aphA* inactivation |
| 897 | AAATCATGCAAAAAAGGAGAGCCTGTCGCTCTCCTGATTTGTGTAGGCTGGAGCTGCTTC | *aphA* inactivation |
| 916 | GCTCTATCTGCGCGAGC | *phnWRSTUV::*Km^R^ verification |
| 917 | GGGTTTGCCAATGTGAAGGTGTATCGTCCGTAATTCCTTTGTGTAGGCTGGAGCTGCTTC | *phnWRSTUV* inactivation |
| 918 | AGGCCGAATAAGCGACAGCGCCATCCGGCAGTTATTTTACCATATGAATATCCTCCTTA | *phnWRSTUV* inactivation |
| 1688 | GGCGCGGACGTGTTACTTAA | *yhjY*-*HA*::Cm^R^ verification |
| 1689 | AGCGATTATTTGTATACCTTGGGGGTGAGCGCCAGGTTTTATCCGTATGATGTTCCTGATTATGCTTAGCATATGAATATCCTCCTTA | *yhjY-HA*::Cm^R^ tagging |
| 1690 | AGCTGTTATCACTGCGTTTCGATTATAATTTTTAAGTTAGTGTAGGCTGGAGCTGCTTC | *yhjY-HA*::Cm^R^ tagging |
| 1691 | GCTACGATTCAGATGGAGTA | *zitB*-*HA*::Cm^R^ verification |
| 1692 | TCATCTGAATCAGACATCGTCCGGGCATGTTCATCACCATATCCGTATGATGTTCCTGATTATGCTTAGCATATGAATATCCTCCTTA | *zitB-HA*::Cm^R^ tagging |
| 1693 | AAACAGCGCGCGGGAGCGAGGATCGCGCGCGCTTTCCCGTTAAGTGTAGGCTGGAGCTGCTTC | *zitB-HA*::Cm^R^ tagging |
| 1694 | GCCGAATATTATTCCACCGG | *cydB-HA*::Cm^R^ verification |
| 1695 | CGTCTTCCGTGGAAAAGTGCGACATGGTGAGGGATATCACTATCCGTATGATGTTCCTGATTATGCTTAGCATATGAATATCCTCCTTA | *cydB-HA*::Cm^R^ tagging |
| 1696 | ATACGACTCGCCGCGACCATTTTTTCACTTTAACCATCAGTGTAGGCTGGAGCTGCTTC | *cydB-HA*::Cm^R^ tagging |
| 1697 | AACATCGTGGTGGTTCAGG | *yciG-HA*::Cm^R^ verification |
| 1698 | GGTCAGAATAGTCACGGCGGACGTAAATCCGATAATTCCTATCCGTATGATGTTCCTGATTATGCTTAGCATATGAATATCCTCCTTA | *yciG-HA*::Cm^R^ tagging |
| 1699 | TGCTTGATAAAAGCATGTGTTATATTTACATTACAGTAAATCGTGTAGGCTGGAGCTGCTTC | *yciG-HA*::Cm^R^ tagging |
| 1700 | ACTGGGTTGATATCGCGAT | *apeE-HA*::Cm^R^ verification |
| 1701 | CAATCAAACCCGTTATAACGTTGGGTTTAGCGCCCGATTTTATCCGTATGATGTTCCTGATTATGCTTAGCATATGAATATCCTCCTTA | *apeE-HA*::Cm^R^ tagging |
| 1702 | CACGAACGAACGGGGTTGGCCCTCCCTGGCGTGTCATCAGTGTAGGCTGGAGCTGCTTC | *apeE-HA*::Cm^R^ tagging |
| 1703 | TGCTACAGACAGCGCTGCCG | *phoN2-HA*::Cm^R^ verification |
| 1704 | GATCTGTCCGCTGCTTACGAGATGGCGAGAAAAACGCGCTATCCGTATGATGTTCCTGATTATGCTTAGCATATGAATATCCTCCTTA | *phoN2-HA*::Cm^R^ tagging |
| 1705 | CATCCCGGCTTACGTTTTATAAGCCGGGTGACAACGTCAGTGTAGGCTGGAGCTGCTTC | *phoN2-HA*::Cm^R^ tagging |

**Cis regulatory elements of PhoB-regulated genes in *Salmonella enterica* strain 14028s (NCBI reference sequence NC_016856.1).**

**Putative promoter(s)** were predicted with SAPPHIRE (7).

Putative Pho boxes on the **positive** and **negative** strands were predicted with MEME Suit (8).

**Primary and secondary** transcription start sites (TSSs) have been experimentally inferred (9, 10). ***** Indicates genes with unknown TSSs.

**Putative translation start/stop sites** were obtained from the genome annotation (11).

**Canonical PhoB-regulated genes in *Salmonella enterica* strain 14028s (NCBI reference sequence NC_016856.1).**

***>phoBR***

gccagcgacaataatggcatccacctgatgggcctgcgcggtctccagcagccagtccagaaaagcctgatgctccgcggcgcggcttttactgtagaaattttg**tcccagatgccagtcagaggtgtggaggatgcgcataattgttcc**atgcaaaaaaagcgtgaacgggattatacacgtcatcccttccatttttgggcgcaatttaccgccggtacacggtaatgcatggtttcaccggtgtcataaatcatcaacatgctgtcaatgccgcc**tttttttttcataaatctgtcataaatctgacgcataatggcgcgG**cattgataactaacgactaacagggcaaatt**ATG**

***>pstSCAB-phoU***

ggtcggtggcgcaatcgccgggggactgtccatgtatttcggttgtacactgatggcgccacacggtggcctctttgtcctggcgatcccgcatgcggtagaacatgtgatgcaatatctgctctcgattgccctgggcacgattgtctgcggcctgatgtacgcgctgttgaaaccgtctgcggttgcgcaaacagtctaattcatctccccctgcaggctggtctggtgtctccccgggcca**gctttttttatttccattgtcatcaaaccgtca**t**a**a**t**c**a**a**g**a**c**a**t**a**taactgtcatcAatttgtcctattttgctcat**c**G**tagcaactcaaacaacgatttaccgaaaccgtgcaggagacatt**ATG**

***>phoE****

ccgttcaaaataagaaataaagaccaaatgaacgttttagcaggactggctccggttgccaacaacctgtacgcgtagcgtgaaattttgttgcgcaggatcagcaagcgtagcggcggaatttgaccattttattaccgcaacaattaaacatatttttttaaaaaaattctcact**ttgtcat**aaat**ctttcat**taccgaacgttaa**aaaccttcctgttttttaccgggtttcccgacaaatcatagcgcg**taattaaaacaggaatggaa**ATG**

***>ugpB***

aatcccggcgacgctccgcctggttgggttacggcatgtgcgccgcccccgagaataatgtcgtacctatccgtgccgtagctcccacttttcttttccggcgct**ttcgctgccttgccatctctctgtcgccttactatcttttttttG**taataaaaaagttatttttctgtcattcgagcatgtcatgttacccccgcgaacataaaacgcgtgatatcgc**gcatcccggcacaaaaagagagataaccgATGatatcgttacgacA**tacagct**TTA**

***>phnS****

ggatgcgccggatgttggctgcgggacttatccggcctaccaatcgtatcgtctcacggctatccggataagcgcagcatcatccggcgtggactgatttatttct**ctttcatcgttttgtcatataagccgtttagcgtaaaaagcaaacct**ggtataggccagaaaatgcaacgtacgctatgaggctattacg**ATG**

***>psiE***

aggcgtatatgtttccgtgccgctggatatcttctcgtccggcccaacccgaagtcgtgcggcaattggctggacgccgctgacgcgtgacggtggtcaacagcttggccgtaagtttggcctgtatgatatgaccagtgacaggagcgtaaatttccgttaatactcaggccggataaggcgtttacggcgctatccggcaaattatcttattgaacaaaatataaacataaa**taaaaaatatagatgcccgtcacatttgtgcgttatacagaaaccT**cgccgcagagaaagagggggctgtt**ATG**

***>ytfK* (*STM14_RS23110*)**

attccttctttatttttttgcaggtgatccgaccactttgggccgatagttaatcatatgtgcgattgatgctttttcccgcaaaggggatgccagtttgcgg**gcgggcgcacacttcctgtgaaaaatgaaggcatatactgagA**aaaatgagctgatgtttag**ataattctgaataactgtaatcaaaaggtaaatata**c**t**t**a**t**g**c**a**c**ActggaaacgacgtagatatggtctatagtcatatgG**cattaaaatttgcgccttaaaactgttgggccgattgtggcatcgcaagggcgtaatactctgcaggagacaaca**ATG**

***>waaH** (*STM14_RS19605*)**

gtgtcattcgccaaccttttttgttagggaaaatctggaaagccgtaaagaattgtcatagacatcaagcattcgtaattgcgctttactcttattttactcgctaacgtcacgctctactctgagttttgtgcttgctttttactgtaaaaattaattatggcggcttaatag**tttcttaatagagccacagtataaaggcagggtaaattaaggttt**ttctggtaatcgtt**ATG**

**Putative PhoB-regulated genes**

***>yiaG* (*STM14_RS19310*)**

ttatctgcatcaatgcttaatttgttattttattgttaatttttgacctgacactgcgataaaa**aacgcggcaagaaagctttc**c**a**g**c**g**g**g**g**a**g**t**g**t**a**a**t**a**t**t**t**t**t**t**t**a**atctttacaattattttct**gaaagacggatataccttcctggatcaaattgttagcgtcctaaaagtcgcgtagcgtaaggcatcgaaacgaatcgatagcgcttcctggcggcgcgtcgccccccacccgcaagtttcccgactattcttaagag**G**cttcgatgcatttcacgatcccgctgtgtgatttacaggagttctca**ATG**

***>yeaG***

ccataattccttattcaaatgttctgcggcaaaagtgtaaccgccaatacctgtaagcccaactattttacacaatgtaaccgtttaatatcaccacaaccgattattttcagatgatcggctgatgatcgttatgtgaccgggaacgctttttctgatatccaccagccttttctacctgatgagttattgatatgtcatcgaaatccac**tgacgcgtacaggcaagttttgcaaatgccatctacgcttaatgt**t**A**agaaggtgtatcaccggacacgttaatcttctgaccaataaaatggcatgagagttgctttttttttccttagcagagacggcgttcagtctacctcttccgggagcctctactattcatatgaacggctcttaacatgtgcgaaaaaacgaaaggatggcatatc**ATG**

***>yncC***

aatggcg**gcattatgtcgccataaaaatgtgcaaattttaaaattgcggctt**tacttatattc**A**tcattcagatcgcggtggggcagcttatctccctcagcggcctgaaccggtcaaaaatctcgatacatgaaggaatgacggagataaaacggttgccataaagaaacctgtattgttgtaaaagatgagaatatcacgcaagtaaattatca**cttttatttatttggccgttcaggcctggattatcttttttacaa**ttaagttccgaaacttcttcgggactcgattgcgatgttgttctgccaagaggtgaaggaaggaaa**ATG**

***>phnO***

gctttacgctggtgcttgatacccaggatgtagcagaagggaagcgctggtttgataaccttgccgcacaagggcaaatcgaaatggactggcaggagaccttctgggcgcagggtttcggcaaggtcagcgatcgtttcggtgtgccgtggatgattaacgtggttaaacatcagcctgccacctaactccccgggaggctcgccctcccccttgccatcagagtgatc**tttggctgcacgttatccgccgtttaa**c**t**g**t**c**a**t**a**g**a**a**t**a**a**a**t**a**atgacggtaGtttgctacagtctggcg**aaag**ATG**

***>ybgS* (*STM14_RS04420*)**

tgtttattccagtgttgccgtacttatcccaatgaatgcaatccctccaatctatctcttcaattaaatagtgtaaacgggcttttacactttgaacggaataatcctggaattcaggaaaaaaac**gcacaaatgtagcgaaaaatgggatctaatctacactttttaactG**taaccactctgtttatgaattattgcaagattctctgctgcgttaacccgcggcggcgaacgctttttatcccttatttgaggatttgactgacacgtgcactgttggaagaggttatccgacatatccaccataacaggagcatctt**ATG**

**>*zitB***

cgacatcaataccaacggcaatactaacagcacgatgcagcatccagacggttcaaccatgaatcatgacggaatgaccaaggatgaagagcataaaaataccatgtgtaaagacggtcgctgcccggatattaataaaaaagtggaaaccggtaatggcgtcaataatgacgtgaataccaaaaccgacggtaccacacagtaatgcaaaaaaaagtctgataacgggagagctttcgctctcctt**tttattttgtcagcgaaaaatcagtatgatgtctacagtattgatG**agaataacaaaggaatgacgtt**ATG**

**>*yciE**_*katN*** ggagaaaaacgaagtacgcgatgcagcgcttatcgccgcggcgcaaaaagtcgagcattacgaaatcgccagctacggcacgctagccaccctggccgagcagctcggctatagcaaagcattaaaactgctcaaagaaaccctcgacgaggaaaaacaaactga**tttaaaacttaccgatttagcagtcagcaatgttaataaaagtgc**tgaacgcaaatcgaaataat**atttttaattatcagcttgcatgattccgattttattatcggagc**agattatcacgcattgaggaatgtaaa**ATG**AATTATACTGAACATTATCATGACTGGCTTCGTGACGCCCATGCCATGGAAAAACAGGCAGAATCGATGCTTGAATCTATGGCCAGCCGTATTGAAAATTATCCTGATATAAAAGCCAGAATTGAACAACATATTAGTGAAACCAAACATCAAATTACCATGCTCGAAGAAGTGTTGGACCGTAATGGCATTTCCCGTTCGGTGTTGAAAGACTCCATGAGTAAAATGGCAGCAATGGGGCAATCTATCGGTGGCATGTTCCCTTCCGATGAAATTGTCAAAGGTTCAATTAGCGGTTATGTTTTCGAGCAGTTCGAAATTGCTTGTTATACCTCCCTGCTGGCGGCGGCAAAAAAAGCTGGCGACACTGCCTCAATTCCGACGATTGAAGCCATTCTGAAAGAAGAAATGCAAATGGCTGACTGGCTTATCAAACATATTCCGCAGACAACGGAACAATTTTTACTGCGATCTGAAGCAGATGGCGTTGAAGCCAAAAAA**TAA**ataataagcagga**ggcaatATGTTTCGACACGTAAAACAACTTCAATATACTGTGCGAG**TGAGCGAACCTAATCCTGGATTAGCGAACCTGCTGCTGGAACAGTTTGGCGGCCCGCAGGGCGAACTGGCGGCCGCCTGCCGCTACTTCACG

**>*STM14_RS08675**_*treZY****

tttgttgacctcaatccgcagacgatggcgccagtcgctttctgggtgctgaatgaagatgaagattttaaaggcggggactacgtagatttccaggaaactgagacgacagcagtgccgctagccgttgagctttgtaagaaaaacccgcagagtgaattaagcaaaataaaagacgaaatcaagaaagaactctcaaaataagagtaaactgatatcaaaacccggcctgtgtgtcgggttttactatttgtgacaccgtcacaaataacatctt**ccatttcttctaccaatcacgtggtattgcactattttcattaga**gccctttcactatatggagaccta**ATG**AAAATTTTACCGCTGGCACTCTTTATCATTCCTTTTCTGGCCGGATGCGGCGCCAATAATACGCCGCCGCAAACACCTATTCCCGGGGAAAAAACCTCTGCCAAATTACGTACCCTGGAAACAGGCGCGGCGGCTATTCAATCCAGACCACCTGTCGATGCCATCAGTACCTACCTTGACGGGTTCCATTTTTATAGCGGTGATAAAAACGGACAGATGGAAGCGCACCATTACGTTACCGTCCTGAACGAAGATGTCATGCAGGCGGTGATTTACGACGGCAATACGAAAAACGCGCGCCTGATGGGGGTGGAGTACATTATCAGCGAACGTTTATTTAAAACGCTTCCTCCCGAGGAGAAAAAACTGTGGCACAGCCACCAGTACGAGGTGAAATCCGGTAGCCTGGTGGCGCCTGGCTTACCGCAGGTCGCTGATAAAGCGTTGATGAGTAAGATTGTTAATACTTATGGCAAGACCTGGCACACCTGGCATACCGACCGGGATAAAACCCTGCCAATGGGTATCCCTGCGCTGATGATGGGCTTTACTGGCGACGGGCAGCTTGACCCCGCGCTGCTGGCCGATCGGGATCGCCGTCTGGGAATTGATACCCAAGCCATTAAACGCGAGCGGCAGGATCTGCCTGAACATCCCGTTGTTAAGGGGGCTAACGCCTGGGAGCAGGGAGAGGTTATCCAGCTACAGCGTGTTCAGGGCTCTGGCGAACACGGGCGAGGCGATACCGCGCACTTCGGTACATCTGAGCAATCCCGACAA**TAA**ttccacggttttacacgcagatacgaaaaagaatgcctgttaagt**gacagcgtcacgtaaagattagccccatagtctatgcttgttctt**cggatgttgtagaggttatgggttaaacggagcgacaa**ATG**

**>*fbaB***

cgaacatcatgaacgacagtttaaaggtcgttttcatttttgaatccttcatgacaggtaaggtacgccatgacaataccatttaataataccttttaaataccattgagcattaattttgaccgatctaacactttttcggacgtga**atcgagtcagcagatagcgccgaaaaaatgcgctaaagttggcaA**aaatggtcggtcagtaccaggcttattgc**G**acccgcaatctgcggggcaggactattcaccttccgataccctatcggaacttacgggagcatagct**ATG**

**>*talA***

agggcgctttgttttaactgctcatccatcttgttgttcctttcacgtatcgttcaaaaagtgacgcggcctggtgcgccgcacattatcaccgtactgcgcaacacaaactttgaaggtctgcaaactatcacgacgcagcgcaagagtttgttatcaactt**ataacggtcatgtta**c**c**t**g**c**t**t**a**t**a**c**c**a**g**c**a**a**t**a**c**c**a**t**g**c**c**t**g**t**ctgctAtgctttttgg**ttatgtagtgtatcaatctttcagaagtgtgaatcaacacactcatctaacactttacttttcaaggagtatttcct**ATG**

**>*cstA***

ttgcctgccgctgcatctggggcggcagaatcaaagctgggaaattacgctctttgacgagcaggggaggcgctgctgcacctgtcgcctgggaacggcggtaatgggatagttctggcaaagtaaaatgcaattgcagcattaacgaagaaaggaagtgatctggttaacactgtaatgtaaaaaatgggt**A**acaatccggttttatcat**gttgttgtgttgttaattacggtaaaggtgttatagaaacaaaatG**taacatctctctggaacacccaaacggacaacaact**ATG**

**>*ybdD** (*STM14_RS03610*)** gcggcaatattccgccgcaatacaccgaatcgcaactcgcgcagttggtattcaataaccgtctggatgccgggctaaccatcttctttatggtggtggtcgtggtgctggcggtcttctctattaagacggcgctggccgctctgaagattgataaaccgacggcgaatgaaacgccgtatgagccgatgccggaaaatgtg**gatgagatcgtgacgcaggcgaaaggcgcgcactaatctttttac**atcaatagccctctccagaataaggagagggcatttgaatgacaggtgacaacg**ATG**

**>*ybdH* (*STM14_RS03615*)**

gcgcgacatgataagccaaccgctcgtgcaaataacgaggaccatcaaaatcagggaagccttgcgataaatttattgcctgatgtttctgcgccagcgcgctcatttgcgtaaaaatcgtggtaccaagatttggcaatttgctttttggaatcagtgggttatttctcattatcttcatacccgccagt**gatgtggttgttgacacactatcacagggtgttagtatttggcaA**tcaagacgtttagatgtctaaatataataacaacggtgagaagaccctaaggacaacacaac**ATG**

**>*ybdR* (*ST14_RS03680*)**

acccaggcagtacatgcaacagatgaatgacgccatcctgttgcgccagaaactcagcgtggcgaatggctttatcgctaagttccatttcaaaaacatcaaccggcataatgattgtcttatacatacccgttac**tccctgttaatcaatactgaatttattcaagcatataataatgttT**ctttc**tgctatttgtctggcaattttaaatattacggcaacctgatgtta**aaaaagttgtgtattttccaggaaaaatcctaaagccgcctgttttgccggcagtctctccgggatagctacccttttatagtcgaacgtgacattcaccgttaccccggaggctgc**ATG**

**>*wrbA***

tgacctccgtcatagattagacatcatattgcataggtaatgaggagtatctcctcgcgcagcgtatgaacgatatcatcagtaacgccgcgtaacattaagcatagtgaaaaacgtcgtagtaggccgcttaccgaaatattctgcaacattggaagccgaatacgcagaataataaccataagaaataaataagttattct**tatatttgggaaaatcaaagcgcttaaaaatgtatcattttgcaa**caaaggtcgccaaattaattattgttataaatcaaagaaatg**gcgctgaaatttgcactcttcacaggagagcatatctttaaataG**acgtagcgaatcgctacacacctgattaatctgaggagtagtacaa**ATG**

**>*STM14_RS08755****

tgtactggggattccggaaatggccgatcccgcgtttaaattccatctggatcgta**tgattacgcttgaactctttgcgtaaagttacaaataatttaataa**aaatcgcttttaatttacaaatagtaacaattaagaaacatcatatctcctcatcgcgccgcgctggcctggttttttatctattctttcactcaatattctaaaaaaacttcaataagttctcaaaagagcgcgtgacgctctgtctcttttttgtgtttttcttcaaatgatatgcgcatgtgaggggtaaa**ATG**

**>*yohF* (*STM14_RS12065*)**

gccagtcaggtagagacgctggcgcagaaaaatcagcatcagcagcagattgagcatgacgcgcagcgtgt**ggtcggtctggcgcaagcgcgctttaatgccggtattattgcggg**ttcccgcgtgagcgaggccaaaatcccggcactgcgcgagcagtgtaacggcttgctgttgcagggccagtggctggatgcctctattcagcttaccagcgcgctgggcggcggttatcattc**ctgatttttccggatgattcagacagggttcctatacttaggctG**tgtatgattcatccggagaggaat**ATG**

**>*lysM* (*STM14_RS15000*)**

ctctttttaaacaatgactaattttgcttgtatggagtgtatatgggtttctggagaattgtatttacgattattttgcctccgctcggcgtacttttaggaaaagggttcggttgggcatttatccttaatatcctgctgacattactcggatatattccggggcttatccacgcattttgggttcagatgcgtcattaattaccggccaccggcggcgcataagc**gccgcttatcaggataacctcattccctctgctacacttttctttG**tgtgtatttttgacaagcgaggtgaat**ATG**

**>*apeE** (*STM14_RS03455*)**

cgggaactggcgctggcacgtttttcgaaagcgcgcagtactatccagtgcaaaataatatgcacgacaactgctgtcagaaaaataataccaaatatcaccaccagcgacgtggtgtgattcatttcaattcccgctaaatcttctacccgggatatcaattcctgcataatctctcctttataaacagcagcctatgatgactgctgaatggcatttatgcaaatcaggacgcctgcgcttcaggcataatcagcaacaga**cgggtaaaagcgcctctggggttatccatacgctatattttatcc**tgcgccctgtaaacgtcgttattttacgcggttccctctttttgaacaggcgccagtccgtcaacaatgaatgaaaactttttaacc**agaatgtttaaaacgattgcaatatctctgtcggataatgattag**caccgtcgtacctatcaacaataacattaaggaacatcgg**ATG**

**>*yciG* (*STM14_RS09540*)**

acgtcaatttcccccgcctttatgtaaagaaatgtcaacaaacaatgcattaacgtttccagggccgcagatagtgacgattgttattaataacaatgcaaagcatttcctgttaaatcgcttattctaaaagaaaatatagaatatattatctctaaaccattttttgtgcgttatttacgcggtaaatcacaactat**ttccgtcaatttgactaatcggtttaaccaactaattttaataggG**tgtcgacacggttaaaaccgattttttcagcaagcaacgagacaggagaaataat**ATG**

***>bapA* (*STM14_RS14685*)**

gaagctggtcaatgttcatatctcatgcatgcctcgaaaatatattctctgtgtctgacgcttatcaatagcaataaataccacaacaagaatatattctttgtttaaaaatttttatagtctaataacacaatacttcaaatgatttttataa**aatttaatattgctgaataccttcacaaaaagtattgtttttatg**ttaaaagagaaaaagaagaaaaaattaaaattcatta**taaaacataaagataaataaaaaattatcatgtattgttagccca**ccctacatctccttaacacaaactccgttatatttcagacgcactaacacctttatcaatag**atcttaatttgcagaaagatttttttctgactatatcatctaatA**cgaaagcactagtcaggcacaaaaaacaaagggttattcggcgagaaaaccagacctccacctacgctcataaaaaagaatatggctacggaaattcatctctcacgatgaacgggaaggctcgtctacgcattttgccctgaacgttgtgcccgttaattaatacacagagcaaatccatcaggagctgattt**ATG**

***>phoN2* (*STM14_RS19030*)**

actgataacgccccgcgcccgcgtgcgtgtccagatagagaaacggtttttctttctcttttagcgactcgatgatcaggctctgaacggtatgtttaaggacgtcggcgtggttgccagcgtgaaagctgtgacgataactgagcatggatgcagatattccgggaagtaaacaagttagccgatagtttaccgcagatccgtagagattaccccagtaccgccatgttgttt**cgcgcgttgtgaaagtattgcaagacgtgttcgtatcatcaaaccGtcaactgc**gcaggccagcatcgcctgtgggcaaggtagtagttttgacctggcgttatttttaatgaaaggaaccata**ATG**

**>*STM14_RS08420***

aacttctgatgacagccagttaaatttatggggttaagttgtctgaatataaagagggcgtgcattcggatttttctacttatttttccgtggtggcttgattgccaaagatttattaccgcttcgctgttgatgt**tttcaaaaaatatatcttccgccatgtgatggtattatgtaaata**atgc**agaaatgaatttgacactgcgcacagggcgactagatttagaactG**tatcacatgatatgagaagacatatcatatttaaaacgcaacatcatcatgaggattatatt**ATG**

**>*ymgE* (*STM14******_RS09875*)**

caaaattttactgatgaattgaccatgcgcccatgaaatacgcagtggaacctgatttttatttaaatcccgtagcacgcctaatatcgctaatggattttttttcaggaactgctcattgtaaccactcacacgaaataacccctgatacgcagactatcgttgggttatcggcatcggtagaaagaacttaatagcgataaataaatttttatgtccgcgagataaat**cagtgttcagaaatgcgacgctcgactttgcctatacttaaaacG**tttttactggaaagagggttcgcaa**ATG**

**>*STM14_RS19935***

tcgcctccgccgtaaacgccgtctcctgcgccaaatcggcaagacatagcccgcgcgtcagccgttccagctctcccagtacgatctcaatacgtctcattacactaccgcttacgaaataaaagagtagtcagaacatacactattctgccacgcaagaaagtgaaggcgcagcgagaaaccgccttcccacttttgcctgacttcaacgccaacggata**gcacgccccgatgagtgaacgtcgcacggtctacacttactct**t**G**aaaaagtgcaaaccgataaggataccgttt**AATG**

***>STnc4130* (sRNA at 3’-end of *STM14_RS23700*)** aaaccag**aaaaatacattgagaaaaagattaatgatattaatataaatgatA**taaaatagtgagcacgcataacataagggcatagtaatatatcataaaatcctttaattactattgcccttgaatagattcactattccacatccttcctttccaacccgatatcccttaactgctcgtcactcattcgtcgcagcgcctgacaggcccgcttacgcagccaccacctttttagcgcccgtctgagctgcacaaagccaatga**ATG**

**>*STM14_RS23900***

gccaagttgctggcggacgatctcgtcccttcccgtaaagtgaaagtggaaacgaccgatggcgtcgtacagctctccggtaccgttgaaactcaggaacaaagcgaccgcgctgaaagcatcgcgaaagccgttgatggcgtaaaaagtgttaaaaacgatctgaaagttcagtaattcgtcgtaattcgtc**ctcccgacgtttgtcgggaggcgtaatgtgcaccacactaaaaatG**tcgcgaatgagtagcctgagcgctcatatttagcggtcgacattaactatggtaaaggagaggctt**ATG**

**Supplemental Figure Legends**

**Figure S1. (A)** Fluorescence from wild-type (14028s) and *phoB* mutant (EG9054) strains of *Salmonella* carrying pP*phoB-gfp*_AAV_ or pP*pstS-gfp*_AAV_. Cultures were propagated to mid-logarithmic phase in MOPS medium containing 1 mM K_2_HPO_4_. Subsequently, cultures were either subjected to a nutritional downshift to MOPS medium lacking K_2_HPO_4_ or any other alternative P source (-P), or maintained in the same medium containing 1 mM K_2_HPO_4_ (+Pi) as control (see Materials and Methods for further experimental details). The maximum GFP expression from *phoB* and *pstS* transcriptional fusions (indicated with a ★) occurs at 35 min following the removal of P. (**B)** Growth curve of wild-type (14028s) or *phoB* (EG9054) *Salmonella* harboring pP*pstS-gfp*_AAV_ subjected to the same nutritional shifts described in (A). **(C)** Viable cell counts of wild-type (14028s) and *phoB* (EG9054) *Salmonella* at the beginning (t_0_) and the end (t_35_) of Pi downshift. *** P < 0.001, paired two-tailed *t* test. In all cases, means ± SDs of at least three independent experiments are shown.

**Figure S2. *Salmonella* PhoB-independent response to P starvation.** Heatmaps depicting fold changes in transcript levels between -P and +Pi treatments for wild-type (14028s) and *phoB* mutant (EG9054) *Salmonella* strains. Graphs show selected transcripts from genes that show no significant changes between wild-type and *phoB* during P starvation (Table S1). Displayed genes are organized by COG categories. Note that gene *RS07905* corresponds to NCBI gene locus tag *STM14_RS07905*.

**Figure S3. *Salmonella* PhoB binding motif.** Sequence logo of the PhoB binding sites. The logo was generated by the MEME algorithm using as input 450-bp upstream of the translation start site of the canonical PhoB-activated operons listed in Table S3 (8).

**Figure S4. Utilization of phosphonoacetic acid as sole P source**. Growth of wild-type *Salmonella* (14028s) in MOPS liquid medium containing (**A**) 22 mM glucose or (**B**) 26 mM glycerol as carbon source and either no P, 1 mM Pi (K_2_HPO_4_) or the indicated concentration of phosphonoacetic acid (PAA) as the sole P source. Growth curves show the means ± SDs of three independent biological replicates and are representative of two independent experiments. (**C**) Growth of wild-type *Klebisiella aerogenes* (ATCC13048), and wild-type (14028s) and *phoB* mutant (EG9054) strains of *Salmonella* on MOPS-glucose-noble agar plate containing 1 mM PAA (left) or 1 mM Pi (right) as the sole P source. Plates were incubated at 37°C during 14-18 h before being imaged. Images are representative of three independent experiments. Dashed lines separate non-contiguous sections of the same plate. Note the presence of P-compound(s) in the noble agar that allows the formation of small *Salmonella* colonies. This is not observed during growth in liquid medium (A-B).

**Figure S5. *Salmonella* genetic requirements for the utilization of glycerol-3-phosphate and nucleotides as the only P source. (A)** Growth of wild-type, *glpT* (MP1735), *ugpBAEC* (MP1736) and *phoB* mutant (EG9054) *Salmonella* strains on MOPS-glucose-noble agar plate containing 0.5 mM of *sn*-glycerol-3-phosphate (Gly 3-P) as the sole P source. **(B)** Growth of wild-type, *ushA2* (MP1779), and *phoB* mutant (EG9054) *Salmonella* strains on MOPS-glucose-noble agar plates containing 0.5 mM of ADP or AMP as sole P source. **(C)** Growth of wild-type, *ushA* (MP1778), *ushA2* (MP1779), *ushA3* (MP1780), *aphA* (MP1785) and *ushA* *ushA2 ushA3* *aphA* (*3ΔushA* *aphA*; MP1796) *Salmonella* strains on MOPS-glucose-noble agar plates containing 0.5 mM of either adenosine monophosphate (AMP), adenosine diphosphate (ADP), adenosine triphosphate (ATP) or guanosine triphosphate (GTP) as the sole P source. In all experiments, plates were incubated at 37°C during 16-18 h. Images are representative of two independent experiments.

**Figure S6. Utilization of G6-P and F6-P by *E. coli*.** Growth from wild-type *Salmonella* (14028s) and *E. coli* (MG1655) on MOPS noble-agar plates containing 1 mM of glucose 6-P (G6-P), fructose 6-P (F6-P), K_2_HPO_4_ (Pi) or no P source. In all experiments, plates were incubated at 37°C during 14-20 h. Images are representative plates of four independent experiments.

**Supplemental Material References**

1. Blattner FR, Plunkett G 3rd, Bloch CA, Perna NT, Burland V, Riley M, et al. 1997. The complete genome sequence of *Escherichia coli* K-12. Science. 277:1453–1462.
2. Fields PI, Swanson RV, Haidaris CG, Heffron F. 1986. Mutants of *Salmonella typhimurium* that cannot survive within the macrophage are avirulent. Proc Natl Acad Sci USA. 83:5189–5193.
3. Pontes MH, Groisman EA. 2018. Protein synthesis controls phosphate homeostasis. Genes and Development. 32:79–92.
4. Datta S, Costantino N, Court DL. 2006. A set of recombineering plasmids for gram-negative bacteria. Gene. 379:109–115.
5. Datsenko KA, Wanner BL. One-step inactivation of chromosomal genes in *Escherichia coli* K-12 using PCR products. 2000. Proc Natl Acad Sci USA. 97:6640–6645.
6. Pontes MH, Groisman EA. 2019. Slow growth determines nonheritable antibiotic resistance in *Salmonella enterica*. Science Signaling. 12:eaax3938.
7. Coppens L, Lavigne R. 2020. SAPPHIRE: a neural network based classifier for σ70 promoter prediction in *Pseudomonas*. BMC Bioinformatics. 21:415.
8. Bailey TL, Johnson J, Grant CE, Noble WS. 2015. The MEME Suite. Nucleic Acids Research. 43:W39–W49.
9. Kröger C, Colgan A, Srikumar S, Händler K, Sivasankaran SK, Hammarlöf DL, et al. 2013. An infection-relevant transcriptomic compendium for *Salmonella enterica* serovar Typhimurium. Cell Host and Microbe. 14:683–695.
10. Srikumar S, Kröger C, Hébrard M, Colgan A, Owen S V, Sivasankaran SK, et al. 2015. RNA-seq brings new insights to the intra-macrophage transcriptome of *Salmonella* Typhimurium. PLoS Pathogens. 11:e1005262.
11. Jarvik T, Smillie C, Groisman EA, Ochman H. 2010.. Short-term signatures of evolutionary change in the *Salmonella enterica* serovar Typhimurium 14028 genome. Journal of Bacteriology. 192:560–567.
